# Exploring the artificial intelligence “Trust paradox”: Evidence from a survey experiment in the United States

**DOI:** 10.1371/journal.pone.0288109

**Published:** 2023-07-18

**Authors:** Sarah Kreps, Julie George, Paul Lushenko, Adi Rao

**Affiliations:** 1 Cornell University Tech Policy Institute, Menlo Park, CA, United States of America; 2 Stanford Center for International Security and Cooperation, Stanford, CA, United States of America; National Taiwan University, TAIWAN

## Abstract

Advances in Artificial Intelligence (AI) are poised to transform society, national defense, and the economy by increasing efficiency, precision, and safety. Yet, widespread adoption within society depends on public trust and willingness to use AI-enabled technologies. In this study, we propose the possibility of an AI “trust paradox,” in which individuals’ willingness to use AI-enabled technologies exceeds their level of trust in these capabilities. We conduct a two-part study to explore the trust paradox. First, we conduct a conjoint analysis, varying different attributes of AI-enabled technologies in different domains—including armed drones, general surgery, police surveillance, self-driving cars, and social media content moderation—to evaluate whether and under what conditions a trust paradox may exist. Second, we use causal mediation analysis in the context of a second survey experiment to help explain why individuals use AI-enabled technologies that they do not trust. We find strong support for the trust paradox, particularly in the area of AI-enabled police surveillance, where the levels of support for its use are both higher than other domains but also significantly exceed trust. We unpack these findings to show that several underlying beliefs help account for public attitudes of support, including the fear of missing out, optimism that future versions of the technology will be more trustworthy, a belief that the benefits of AI-enabled technologies outweigh the risks, and calculation that AI-enabled technologies yield efficiency gains. Our findings have important implications for the integration of AI-enabled technologies in multiple settings.

## Introduction

In August 2022, *The New York Times* observed “we’re in a golden age of progress in artificial intelligence (AI). It’s time to start taking its potential and risks seriously” [1]. Amid these rapid developments in AI, discussion of human agency is often absent. This oversight is puzzling given that individuals will ultimately be responsible for whether AI-enabled technologies diffuse widely across society or confront resistance. Regulators will have some role to play, and researchers have studied local officials’ reactions to AI-enabled technologies precisely because they will make important decisions about if, when, and how to use these capabilities [2]. Notwithstanding these insights, we contend that public attitudes are crucial to societal adoption of AI-enabled technologies. If the public is hesitant, demonstrating reluctance to adopt these technologies, then it will pressure policymakers to impose restrictions on research and development [3].

In this study, we research public attitudes both toward AI-enabled technologies across various domains and the basis for those attitudes. Existing studies about public attitudes toward emerging technologies have tended to focus on levels of trust, which is defined as the “willingness to make oneself vulnerable” to a capability “based on a judgment of similarity of intentions or values” [4]. Research on nanotechnology focuses on the public’s degree of trust in the capability to minimize risks to humans [5, 6], as does research on genetically modified organisms [7] and online shopping [8]. Studies on AI-enabled technologies are no different, with researchers preferring to use trust as the dependent variable [9]. This focus on trust may not be misguided. In the context of nuclear energy, for instance, researchers have studied public trust, in part because previous work shows a strong relationship between low levels of trust and low public support for nuclear power [10]. Researchers have also shown that public trust for autonomous vehicles correlates with greater levels of acceptance and adoption [11], which is similar for public attitudes regarding mobile banking [12].

We draw on this scholarship to suggest and test the possibility of a “trust paradox,” which we define as the public’s puzzling willingness to support or use AI-enabled technologies that they do not trust. Such a dynamic is well documented in the social media space [13–15], where the public heavily uses social media despite expressing concerns about data privacy, content moderation, and misinformation. Why would the public support the use of AI-enabled technologies it does not trust? In addressing this question, we advance five hypotheses that help explain the puzzle of why individuals support the use of AI-enabled technologies despite having lower levels of trust: the “fear of missing out” (FOMO); a cost-benefit analysis wherein individuals see risk but are persuaded by the potential benefits; assessments about the absence of efficient alternatives; optimism about the future development of more trustworthy technology; and, transparency about the nature of technology.

First, one potential explanation for varying levels of support and trust of AI-enabled technologies centers on FOMO. The literature on consumer psychology points to FOMO as a powerful factor that can influence people to embrace an experience or purchase a product, even if they believe that doing so can be self-defeating. This FOMO mechanism has been cited as the reason why individuals often overuse smartphones, sleep too little, or abuse drugs, despite recognizing that such behavior contradicts privately-held beliefs or values [16]. We apply this insight, which is related to the implications of other social-psychological considerations such as status [17] and reputation [18] on personal behavior [19], to AI. We argue that FOMO may explain why individuals support the use of AI-enabled technologies that they do not trust. People may believe that if they do not use a certain AI-enabled technology, others will, resulting in feelings of anxiety or loss because they are somehow missing out on seemingly popular activities.

Second, the public’s trust paradox for AI-enabled technologies may also result from a calculation that while these technologies introduce risk, they also provide more benefits overall. Indeed, people may rely on AI-enabled technologies not so much because they trust these capabilities but because they perceive that the anticipated benefits [20] of adopting AI-enabled technologies will exceed the expected costs [21] of doing so. This amounts to an expected utility calculation about the benefits and costs of behavior characteristic of the risk management [3] of new technologies, in which the risks are assessed relative to overall benefits and adopted if the latter outweighs the former. In the context of AI-enabled technologies, this calculation might also help explain individuals’ support of new capabilities that they do not trust, reflecting a belief that they stand to enjoy benefits that offset their perception of costs in the face of distrust. While individuals may not trust an AI-enabled technology, they may still understand it as conferring improvements over a human-driven alternative.

Third, individuals might support the use of technologies at rates that exceed trust if they see few economically viable alternatives. For example, AI-enabled technologies can be used as a substitute for human labor across a range of tasks, especially through automation [22]. Brynjolfsson and McAfee [23] have traced the way that automation has increasingly added efficiencies to the economy, performing medical diagnoses, replacing humans on assembly lines [22, 24–27], and carrying loads too heavy for humans. In other examples, accounting, sales, and trading-related tasks are being replaced by AI [28]. These developments create thorny issues of trust and privacy, but also might seem inevitable, as emerging technologies in the past have also remade societies and economies [26, 27]. Individuals, then, might share these concerns but nonetheless acknowledge that AI-enabled technology can perform particular jobs better than humans, and potentially, replace these human jobs.

Fourth, we posit that technological optimism may be another explanation. In this case, individuals may believe that even if they face risks in the present, future iterations of an AI-enabled technology will improve in ways that minimize such potential harms, which is consistent with technological improvements in the past [29]. A previous study of individual technology use showed that whereas many individuals believe that digital capitalism currently disadvantages large swaths of society as well as erodes trust in democratic institutions, they also think future technology will provide solutions to these challenges through the protection of speech and empowerment of citizens [30]. Based on these findings, individuals might not trust AI-enabled technologies now, but have confidence that these capabilities will improve over time. Such technological optimism may encourage them to adopt AI-enabled technologies in the short-term given the promise of longer-term improvements.

Finally, individuals may not trust AI-enabled technologies but support their use if there is transparency or explanation for how these technologies are used. One of the reasons people have distrusted AI is because the enabling algorithm is perceived as a “black box.” The lack of explanation for coding decisions as well as datasets to train the enabling algorithms creates the potential for bias in AI [31]. This helps explain why Twitter’s CEO, Elon Musk, recently stated “transparency is key to trust” [32], echoing Putnam’s findings that trust is integral to democratic society [33]. Thus, Musk promised to better communicate the social media platform’s data management protocols because of concerns that Twitter “interfered” in the 2020 U.S. presidential election [34]. In the context of AI-enabled technologies, then, the expectation of transparency may account for the public’s willingness to use these systems that they otherwise distrust [35].

## Materials and methods

To study the potential differences between public attitudes in terms of support and trust, both within and across different applications of AI, we constructed a two-part empirical study conducted on a representative sample of 1,008 U.S. citizens. Our project received an exempt status from the Cornell University’s Institutional Review Board for Human Subject Research (IRB Protocol #2004009569), and we obtained consent from respondents electronically. The form of consent obtained was electronic. No minors were included in this study. Subjects were properly instructed and have indicated that they consent to participate in our study by electronically approving the appropriate informed consent. First, although observational evidence points to a trust paradox in some contexts, we seek to establish whether this is a generalized phenomenon across AI-enabled technologies by fielding a conjoint survey that assesses perceptions of support and trust in these technologies.

Second, building on the five mechanisms outlined above, we explore the reasons why individuals exhibit differences in support and trust using causal mediation analysis. We administered the study from October 7–21, 2022, via Lucid. Lucid uses a quota sampling protocol to produce samples that correspond to U.S. demographics in terms of age, gender, race, and location. Existing research shows that this sampling protocol produces experimental effects that largely mirror those found with probability-based sampling [36]. We present summary statistics in the Supplementary Information.

**Fig 1:**
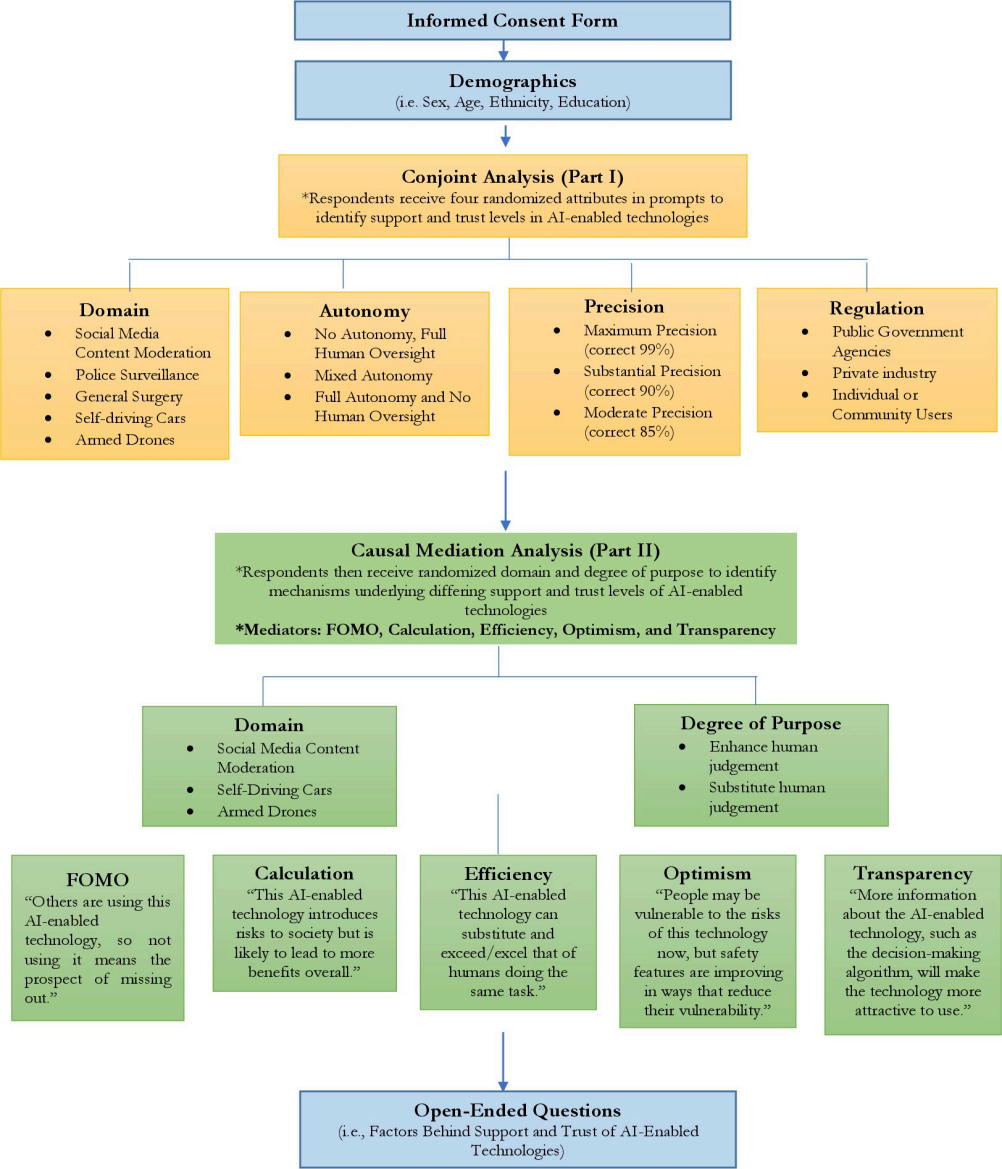
Survey flowchart of conjoint and causal mediation analyses via lucid (October 7–21, 2022).

As Fig 1 shows, respondents first participated in a conjoint experiment to investigate the potential gap between support and trust in emerging technology. Marketing surveys commonly use conjoint surveys [37] because they enable researchers to vary a number of attributes and assess how levels of those attributes affect individual choice. Research also finds that conjoint surveys help reduce social desirability bias among respondents, in which they are encouraged to answer in a certain way or do so because they feel obligated [38]. In adopting this approach, we ensure to fulfill a key assumption for the orthogonality of attributes in expectation, which enables us to differentiate complex treatment effects into their constituent parts [39, 40]. In our case, we are able to assess multiple factors that could plausibly relate to the anticipated effects of AI attributes, either discretely or when interacted with each other, on respondents’ preference formation.

Initially, we informed respondents that they would be presented with a series of hypothetical applications of AI-enabled technologies. We varied four different attributes, outlined in Table 1, along with their respective levels [41]. Research suggests that hypothetical but realistic combinations of attributes can help enhance the external validity of survey results by calibrating contextual detail with experimental control [42, 43].

**Table 1:** Considered attributes and attribute levels for artificial intelligence applications.

AI Attributes	Levels
Domain	Cars Armed drones General surgery Social media content moderation Police surveillance

Degree of autonomy	Fully autonomous (no human in the loop) Mixed-initiative (on-/off switch between human and machine) Manual (no autonomy)

Precision	Maximum precision—a model that produces up to no false positives, and is correct 99% of the time. Substantial precision—a model that produces 10% false positives, or in other words, is correct 90% of the time. Moderate precision—a model that produces 15% false positives, or in other words, is correct 85% of the time.

Regulation	Public government agencies Private industry Individual or community users

In the first part of our experiment, the conjoint survey, we consider different domains in which AI operates. Research on attitudes toward AI typically focuses on one domain, such as autonomous vehicles [44]. We advance the literature on individual preferences for AI-enabled technologies by considering how respondents may have different perceptions of risk across domains, which allows us to understand—theoretically and empirically—how receptiveness to AI varies depending on context. We select domains where AI is already making inroads, including autonomous vehicles, armed drones, general surgery, social media content moderation, and police surveillance. In doing so, we draw on categories identified in the *One Hundred Year Study of Artificial Intelligence* published by Stanford University, which studies how AI will affect society in the future [45].

Within the conjoint survey we also evaluate the degree of autonomy. Drawing on the autonomy literature [46–48], we locate a range of autonomy that is bookended by extreme variations and includes a hybrid form of automation as well. At one extreme, we present respondents a form of manual autonomy that is characterized by human control with no machine assistance or involvement. At the other extreme, we present respondents with a form of full autonomy where the machine has full responsibility and control over decision-making in these settings. Splitting the difference between these two extreme variations in autonomy is mixed-initiative autonomy. Respondents presented with this level of autonomy are informed that the application can toggle between human and machine control in these settings.

We then vary the degree of algorithmic precision. Model performance is often a function of convergence between the algorithm and truth. In a context of natural language models, for example, algorithmic detection of AI-generated text is measured based on the percentage of text examples that the algorithm correctly identified as AI versus human generated [47]. Social media platforms such as Facebook advertise that they remove 99.8% of terrorism-related content before users flag it, and 97% for hate speech that violates community standards [49]. Algorithms trained to detect cancer are measured in terms of accuracy of diagnoses, with some neural networks reaching 99.8% [50]. In our study, we use language concerning the precision of algorithms, which refers to the percentage of the results which are relevant. Designing an algorithm for precision yields confident diagnoses. In the context of oncology, this means that an algorithm correctly diagnoses someone with cancer while avoiding Type II errors or false positives. A model that has the precision of 0.95 when predicting a diagnosis, then, would be correct 95% of the time. How individuals view these precision rates is likely to be a function of the domain in which algorithms operate, interacting with variables such as the controllability to affect what they deem to be “sufficiently high” levels of precision.

We additionally consider the locus of regulation. One approach is for government agencies to regulate the way that businesses use algorithms by enforcing legislation such as the Credit Reporting Act. A second option is for a private firm that developed the software or platform to regulate it, similar to the approach adopted by Facebook and Twitter. A third approach is public-private collaboration where public agencies direct private firms to alter their behavior. The Centers for Disease Control often request that Twitter flag particular content relating to COVID-19 vaccines as misinformation [51]. Finally, individuals, users, or communities engaging on a particular social media platform may self-regulate, which is consistent with Reddit’s approach to content management. Reddit communities adopt this approach in terms of creators developing guidelines for user behavior and communities regulating on subreddits [52].

Taken together, the conjoint task randomly varied four attributes and levels therein, yielding 135 unique scenarios for an AI-enabled technology, which a recent study shows is well within the tolerance level for quality responses [53]. Given our within-subject survey design, we presented respondents with four randomly assigned choice sets resulting in over 5,000 observations. After reading each scenario, we then asked respondents two questions that define our key dependent variables. First, we ask whether respondents support the use of AI in these settings, using a 5-point Likert scale to gauge their attitudes (1 corresponds to “strongly disagree” and 5 to “strongly agree”). To gain leverage over a possible trust paradox where respondents do not trust an AI-enabled technology but nevertheless support its adoption, we ask subjects if they trust the use of AI for these purposes as well. We study trust as one of our outcome variables rather than a willingness to use AI-enabled technologies for several reasons. Measuring trust is consistent with previous research for emerging technologies and more importantly, is a direct test of public attitudes. Measuring respondents’ willingness to use AI-enabled technologies, on the other hand, may increase the potential for confirmation bias regarding these capabilities.

To analyze our data, we first evaluated the potential for a trust paradox, initially calculating marginal means for the degree of support and trust for each AI-attribute level in a manner similar to other conjoint studies in political science [54]. Doing so allows us to identify the “trust paradox,” which we do by determining the statistical difference between public support and trust for each AI-attribute level. We then calculate the average marginal component effect (ACME) per attribute level, which is generated by our conjoint design, in a regression framework. The ACME represents the mean difference in a respondents’ level of support and trust for an AI-enabled technology when comparing two attribute values averaged across all combinations of other AI-enabled technology attribute values. Based on the randomization of our attribute-levels, we assume that these ACMEs are nonparametric, meaning they are not the result of an underlying distribution of data [55–57]. We also include several control variables in our regression framework based on existing research that suggests these may have potentially important mediating effects on public attitudes for AI-enabled technologies. Specifically, we are interested in how differences in age, gender, race, and political ideology may shape respondents’ support and trust for AI-enabled technologies [58, 59].

Beyond establishing whether a trust paradox exists, we then presented respondents with another survey experiment to assess the potential microfoundations, or underlying values and beliefs developed in the introduction, that shape degrees of support and trust in AI-enabled technology. This consisted of a 3x2 factorial and between subject survey including six treatments and a control group. Those randomly assigned to the control group only learned that “In recent years, advancements in Artificial-Intelligence (AI) have led to the emergence of new technologies.” From the five domains we studied in our conjoint survey, we selected three that scholars broadly recognize as the most “hotly debated” [45]: armed drones, social-media content moderation, and driverless vehicles. These three domains also capture the use of AI-enabled technologies in various settings, such as in conflict (armed drones), across society (driverless cars), and in the online space (social media content moderation), which further allows us to investigate potential differences in support and trust.

For each technology, the experimental groups also varied two intended purposes, whether to enhance human judgment or provide a substitute for human judgment, which draws on debates about “complementing” versus “replacing” human systems. One argument suggests that AI-enabled technologies have different cognitive qualities than biological systems—including a set of mental models about ethics and empathy—and should be viewed less as a replacement for human intelligence but rather as a partner to humans [60].

After reading their vignettes, we asked respondents to gauge their support, trust, and understanding for AI-enabled technologies with varying purposes. Previous research on mediators in terms of AI-enabled technologies has focused largely on the implications of affect, such as anger, on degrees of public support [61]. Rather, we cast a wider net of potential mediators, building on arguments about trust and support in the literature on emerging technology discussed in the introduction. Specifically, we draw on the five mechanisms outlined in the introduction to develop corresponding statements and ask individuals their degree of agreement or disagreement with each of these statements, using a 5-point Likert scale to capture their feedback (1 corresponds to “strongly disagree” and 5 to “strongly agree). For the FOMO mechanism, for example, we asked subjects to respond to the following statement: “Others are using this AI-enabled technology, so not using it means the prospect of missing out” [16]. We repeated this approach for the other potential mediators, as outlined above in Fig 1.

Based on the responses to these questions, we then carried out causal mediation analysis. This method shows the complete causal chain for the effect of an independent variable on a mediator and the effect of a mediator on the dependent variable [62, 63]. Though we ensure to fulfill key assumptions to operationalize this method, namely randomizing the order of survey questions for respondents across all groups [64], causal mediation analysis is sometimes criticized for failing to account for confounding variables, even in an experimental setting that researchers usually champion for resolving stochastic error. Miles argues that confounders “cannot be eliminated even in a well-controlled randomized experiment” [65], causing Simonsohn to contend that “we over-estimate the mediators” [66]. In recognition of these valid concerns, we draw on previous studies that attempt to adjudicate public attitudes for AI-enabled technologies. These studies adopt what Imai et al. refer to as the “sequential ignorability assumption,” whereby both possible pretreatment confounders and treatment assignment are assumed to be statistically independent from the potential outcomes and mediators [67]. Additionally, we opt not to inductively derive possible mediators from respondents’ answers to open-ended questions, as other researchers have done [68], given the possibility of bias [69].

## Results

**Fig 2:**
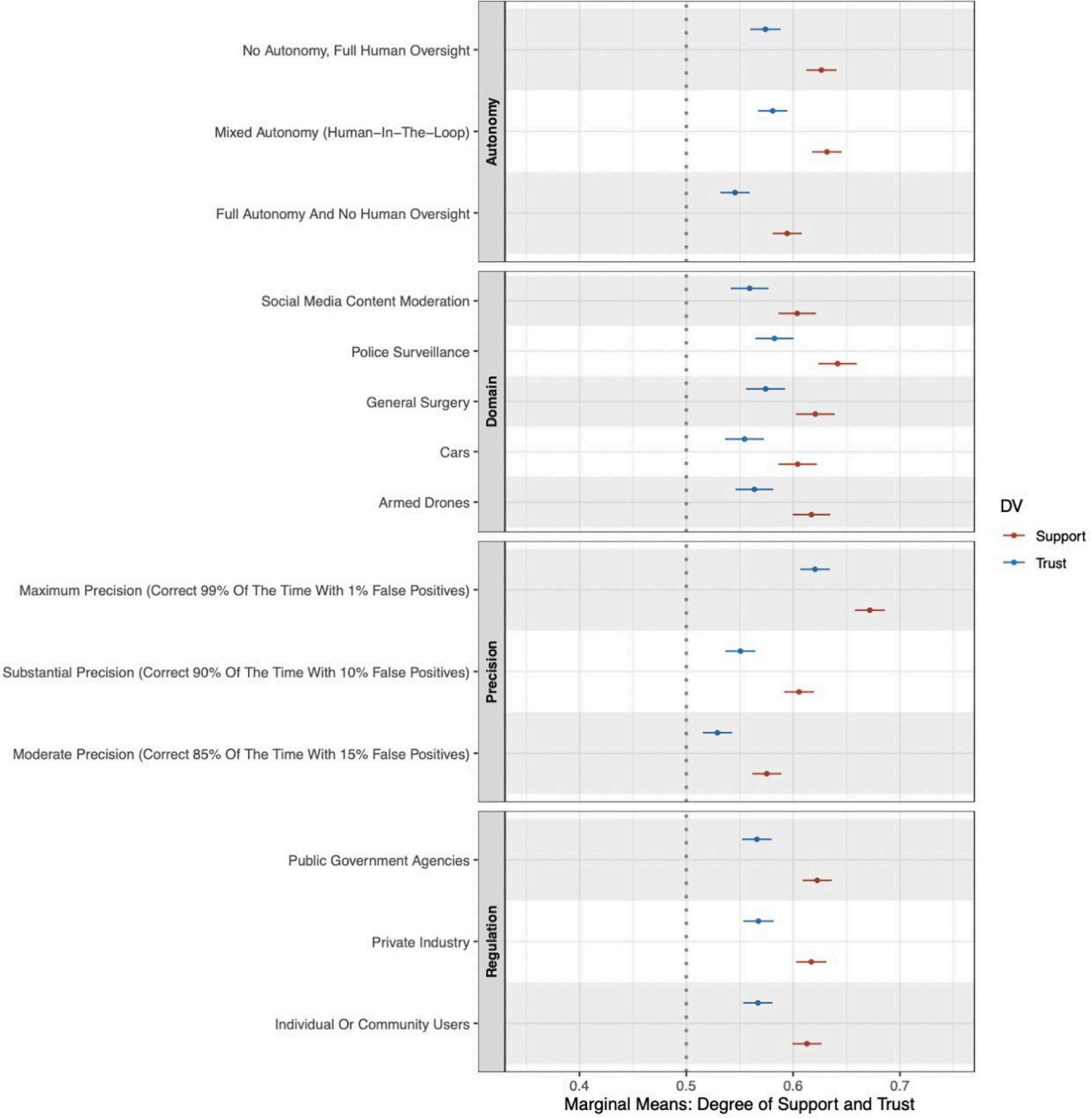
Support and trust marginal means by attribute levels. Caption: AI-enabled technology attributes and support or trust for the technology. The figure shows the marginal means for each attribute value for support or trust in the hypothetical AI-enabled technology, as indicated by respondents after reading their assigned scenarios, on a 5-point scale (rescaled to vary from 0 to 1). Estimates are based on Table 2. Error bars represent 95% CIs around each point estimate.

We report the results of our conjoint analysis assessing the trust paradox and then the experimental treatments evaluating the underlying mechanisms of public attitudes. Fig 2 presents the marginal means for each attribute and level for public attitudes defined in terms of support and trust. Our analysis of the data points to statistically significant differences between the trust in an AI-enabled technology and the support of its use across certain domains, and at different autonomy and precision levels. Below, we discuss the results of our conjoint analysis and then model the data within a regression framework.

Fig 2 shows significant gaps between trust and support across attributes, validating the notion of a trust paradox in which individuals’ support for use exceeds that of their trust. Below, we report the marginal mean sizes and statistical significance of gaps between support and trust per attribute. These differences reflect around a 5% difference in levels of support and trust. In terms of domain, we find that the trust paradox is largest for police surveillance (-0.059 points; *p* < 0.001), followed by drones (-0.054 points; *p* < 0.001), cars (-0.050 points; *p* < 0.001), general surgery (-0.047 points; *p* < 0.001), and social media content moderation (-0.045 points; *p* < 0.001). We use a second survey experiment in conjunction with causal mediation analysis to unpack these results, which we discuss in the section following. Turning to autonomy, we see a significant decrease in trust relative to support for full human agency (-0.052; *p* < 0.001), mixed autonomy (-0.051 points; *p* <0.001), and full autonomy (-0.049 points; *p* < 0.001). While the trust paradox is equally consistent for precision across the three—85%, 90%, and 95%—levels, we find the gap is highest for the middle tier (-0.055 points; *p* < 0.001). Lastly, in terms of regulatory agent, we also find gaps between support and trust, whether the agent is an individual or community users (-0.046 points; *p* < 0.001); private industry regulation (-0.050 points; *p* < 0.001); and, for public government agencies, trust is also lower than support (-0.057 points; *p* < 0.001).

Next, we generated OLS regressions based on whether individuals support and trust the AI-enabled technology. We present our results in Table 2. Model 1 shows support for the use of the technology as the dependent variable. Model 2 replicates these results while using trust as the dependent variable. Given the structure of our data, that is, our use of categorical variables, we produce these results by using a referent level within each attribute, which are identified in the table notes below.

**Table 2:** Attributes and public preferences on ai-enabled technologies.

	No Controls		With Controls	
	Support	Trust	Support	Trust
(Intercept)	3.265***	3.096***	3.392***	3.201***
	(0.063)	(0.064)	(0.132)	(0.133)
Armed drones	0.052	0.037	0.054	0.039
	(0.055)	(0.054)	(0.054)	(0.053)
General surgery	0.067	0.079	0.080	0.093+
	(0.053)	(0.054)	(0.051)	(0.052)
Police surveillance	0.150**	0.113*	0.155**	0.117*
	(0.055)	(0.054)	(0.054)	(0.053)
Social media content moderation	-0.002	0.019	-0.011	0.012
	(0.055)	(0.054)	(0.053)	(0.052)
Full autonomy and no human oversight	-0.128**	-0.114**	-0.132**	-0.117**
	(0.043)	(0.042)	(0.042)	(0.042)
Mixed autonomy (human-in-the-loop)	0.021	0.027	0.024	0.031
	(0.041)	(0.041)	(0.040)	(0.040)
Maximum precision (correct 99% of the time with 1% false positives)	0.386***	0.366***	0.394***	0.374***
	(0.041)	(0.042)	(0.040)	(0.041)
Substantial precision (correct 90% of the time with 10% false positives)	0.122**	0.086*	0.108**	0.074+
	(0.040)	(0.040)	(0.039)	(0.039)
Private industry	0.016	0.002	0.015	0.001
	(0.039)	(0.040)	(0.038)	(0.039)
Public government agencies	0.038	-0.004	0.039	-0.004
	(0.041)	(0.041)	(0.041)	(0.041)
Male			0.188**	0.213***
			(0.057)	(0.057)
Conservatism			-0.041*	-0.037*
			(0.018)	(0.019)
Income			0.040+	0.027
			(0.022)	(0.022)
Education			0.069**	0.067**
			(0.023)	(0.023)
White			-0.094	-0.074
			(0.062)	(0.065)
Age			-0.008***	-0.008***
			(0.002)	(0.002)
Num.Obs.	5040	5040	5040	5040
R2	0.024	0.021	0.071	0.062
R2 Adj.	0.022	0.019	0.068	0.059
RMSE	1.16	1.16	1.13	1.14
Std.Errors	by: id	by: id	by: id	by: id

+ p < 0.1

* p < 0.05

** p < 0.01

*** p < 0.001

Caption: Conjoint average marginal component effects (AMCE) per attribute level. We use cars, human only autonomy, 85% precision, and community and individual regulations as referents for domain, autonomy, precision, and regulator respectively. The dependent variable is a 5-point Likert scale.

Our analysis suggests that full autonomy significantly reduces respondents’ support (β = -0.132, *p* < 0.01) and trust (β = -0.117, *p* < 0.01) for an AI-enabled technology. We note, however, that there is no effect when comparing the fully human system referent level to mixed autonomy. In terms of domain, we see that, relative to the driverless cars referent level, police surveillance significantly increases respondents’ support (β = 0.155, *p* < 0.01) and trust (β = 0.117, *p* < 0.01). We do not detect an effect for any other domain. These results, however, are moderated by the precision. We find that 99% accuracy favorably shapes public support (β = 0.394, *p* < 0.001) and trust (β = 0.374, *p* < 0.001). For 90% accuracy, our analysis also shows that support (β = 0.108, *p* < 0.01) is significant yet trust (β = 0.074, *p* < 0.05) is not. Similarly, we do not find that variation in the regulatory agent shapes public attitudes of support and trust. While respondents might care that *some* regulations exist, they are otherwise agnostic to how different patterns of regulation may affect AI-enabled technologies.

We also assess how different demographic variables may impact levels of support and trust, including race, age, gender, and education. We do not find significant differences in support or trust for AI-enabled technologies on the basis of race. That we do not detect stronger effects is somewhat surprising given the research on the range of biases that AI can have for communities of color, whether in terms of algorithms that discriminate against black patients on transplant lists [70], in policing [71], and marketing [72]. Black individuals have been found to express more distrust for AI-based facial recognition technology, perhaps because of the negative association with biased algorithms [73]. Our findings may point to less distrust for algorithms than human judgment when it comes to policing but we urge additional study to probe these attitudes more fully.

We do see, however, an association for age. Being older is associated with less support (β = -0.008, *p* < 0.001) and trust (β = -0.008, *p* < 0.001) in AI-enabled technologies. This is consistent with public opinion polling [74] that suggests millennials use technology more than older generations, with 93% of millennials (23–38 years old) owning smartphones compared to older people (74–91 years old). There may also be differences based on gender, with men more likely than women to support (β = 0.188, *p* < 0.001) and trust (β = 0.213, *p* < 0.001) the use of AI-enabled technologies. These findings reflect earlier research for a gender-bias in terms of AI, with men more likely to support and trust emerging technologies than women [75, 76]. Education is also positively associated with both support (β = 0.069, *p* < 0.01) and trust (β = 0.067, *p* < 0.01); however, conservatism appears to be negatively associated with support (β = -0.041, *p* < 0.05) and trust (β = 0.037, *p* < 0.05). Similar to our finding for race, we approach any interpretation of non-randomized variables with caution—we do, however, present interesting avenues for future research along these domains.

### Examining the basis for support

The initial part of our study found a significant trust paradox, with individuals supporting the use of technologies at substantially higher rates (e.g., the difference of β = 0.155 and β = 0.117 for support and trust, respectively, for the association with police surveillance) than their levels of trust. This corroborates our theoretical hunch but does not necessarily provide clues as to why the public would demonstrate high levels of support for technologies they do not necessarily trust. To probe the basis of that paradox, we conducted a second part of the study, which investigated the factors that mediate the relationship between the technology and public attitudes of support or trust.

We also investigated the extent to which respondents understand the AI-enabled technologies, especially since AI can be difficult to visualize or comprehend. Indeed, “black box AI” has been used to describe an AI system whose inputs and operations are not visible to users [77]. Including questions regarding the understanding of AI-enabled technologies in different domains allows us to proxy for respondents’ self-assessed knowledge as well as determine how this relates to levels of support and trust. These questions on understanding were asked after the questions on support and trust. It is possible, then, that respondents may demonstrate degrees of understanding that are at odds with their levels of support and trust, suggesting important implications for the regulation of technologies.

**Fig 3:**
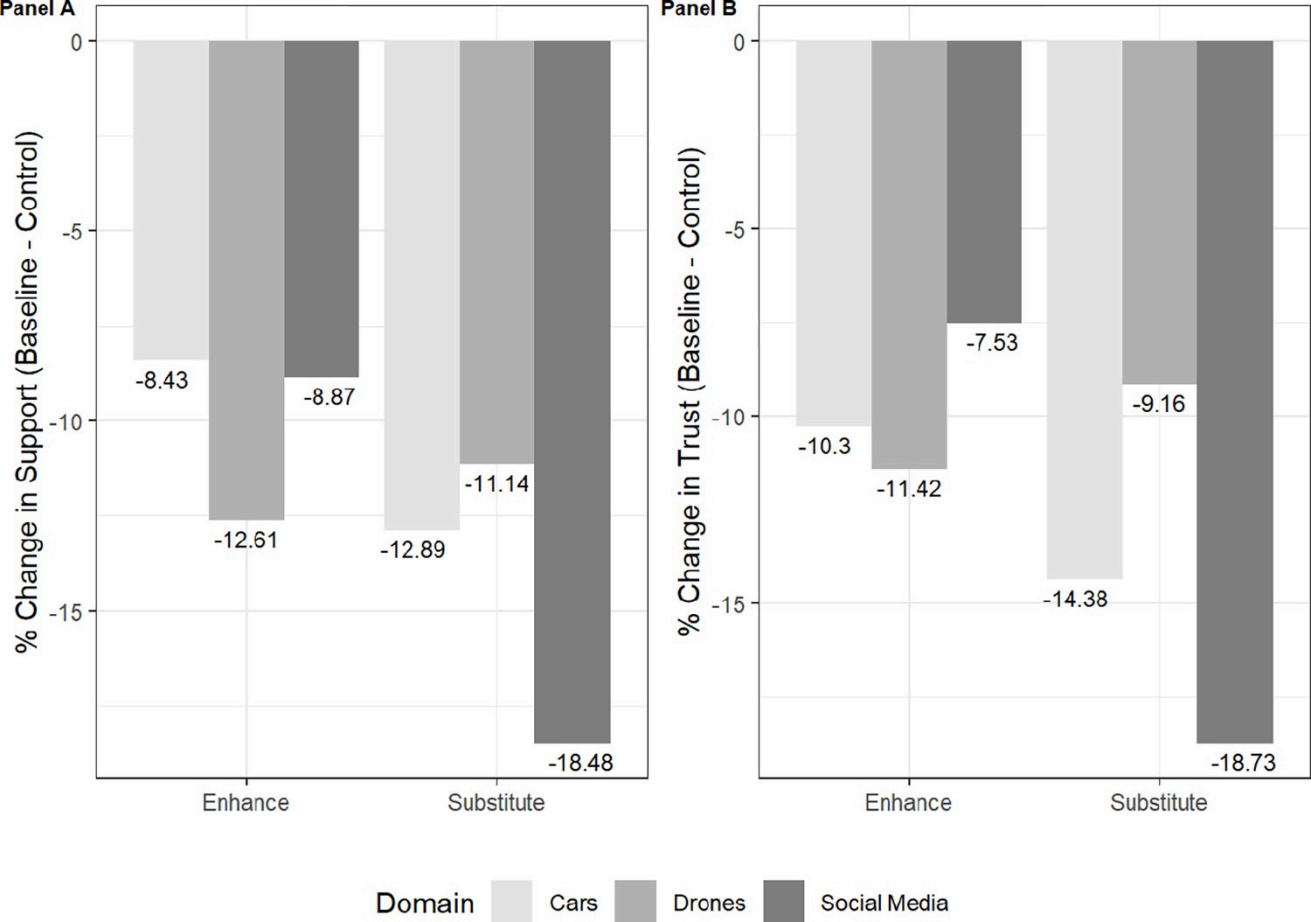
Support (Panel A) and Trust (Panel B) for Treatment Conditions Relative to the Control. *Caption*: Values represent changes in levels of support and trust for AI-enabled technologies by treatment conditions compared to the control condition. Values are negative because levels of support and trust drop compared to the control or baseline group.

This second experiment is not directly comparable to our first, meaning we do not directly study the “trust paradox” identified above. Rather, we use this second experiment to gain insights into the role that underlying beliefs may play in shaping overall attitudes for support and trust for AI-enabled technologies, which we show can and often do deviate. In doing so, we replicate an approach adopted by other scholars studying emerging technologies [78, 79]. Fig 3 shows the decline in support (Panel A) and trust (Panel B) for each domain of AI-enabled technology and depending on whether the technology enhances or substitutes for human judgment, relative to the control condition, which referenced generic advancements in AI leading to new technologies. As the figure shows, the domain of social media reduced trust and support the most of any treatment condition in terms of enhance and substitute for human judgment, and by nearly the same margin. Social media content moderation to substitute for human judgment decreased trust and support by 18.73% (*p* < 0.001) and 18.48% (*p* < 0.001), respectively, relative to the control group. The difference between levels of support for social media content moderation that enhances rather than substitutes for human judgment is also statistically significant (8.87%, *p* < 0.05), which is similar to changes in levels of trust (7.53%, *p* < 0.05).

Drones and cars, on the other hand, decreased trust and support at lower levels relative to the control and across both types of AI purposes—enhance human judgment and substitute for human judgment—and at statistically-significant levels (*p* < 0.001). Whereas the public shows less trust than support for cars in terms of AI-enabled technology that both enhances and substitutes for human judgment, the results are reversed for drones. The public shows more trust for drones relative to the control group, in terms of AI that both enhances and substitutes for human judgment, than it does for support. At the same time, the public is more apt to trust and support drones—relative to the control group—when AI is used to substitute for human judgment rather than enhance human judgment. This may suggest that people have limited knowledge of how drones work, which still require a “human-in-the-loop” to authorize strikes; believe that drones incorporate more AI than they actually do; or, are more receptive to fully autonomous drones than most scholars believe [80].

**Fig 4:**
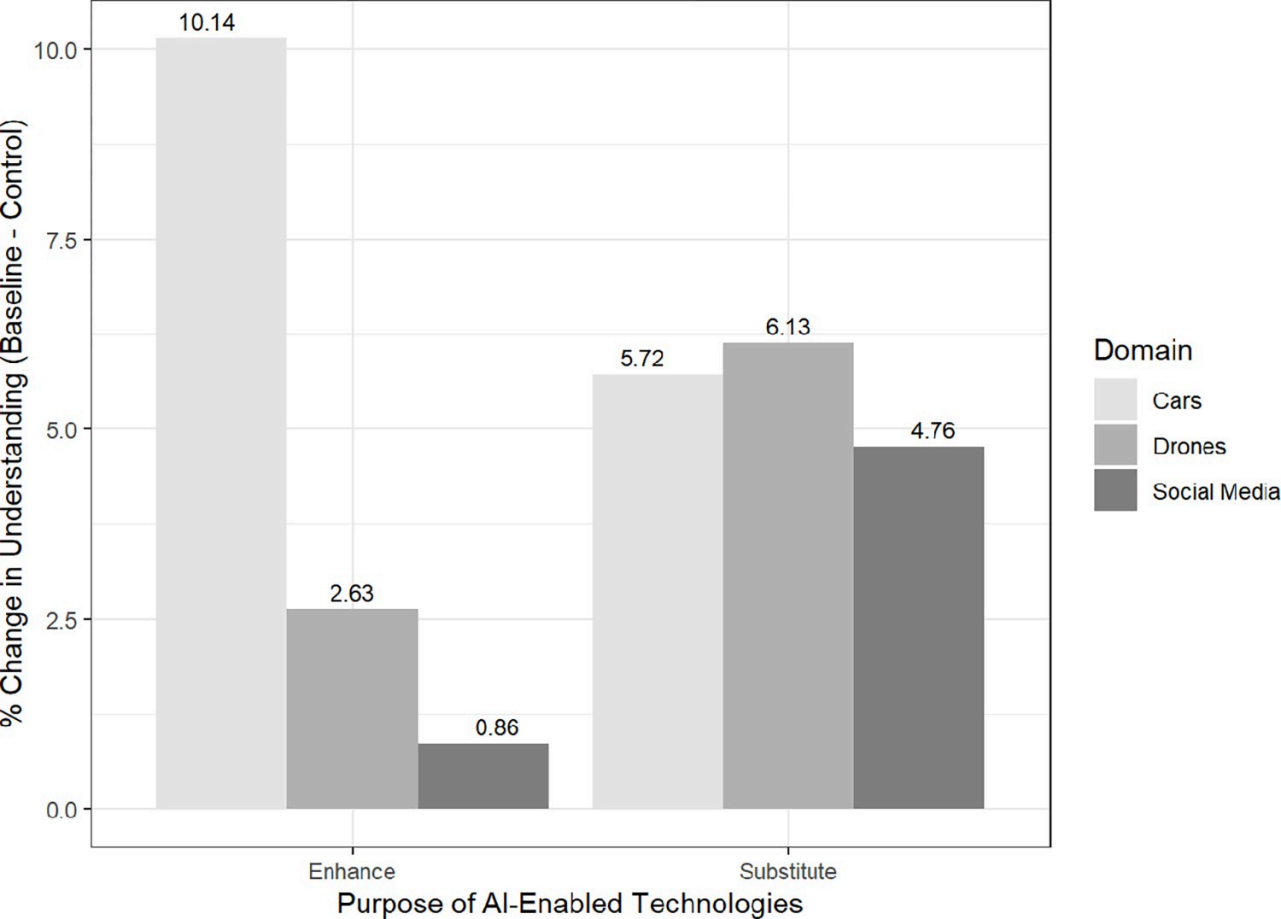
Change in understanding for treatment conditions relative to the control. *Caption*: Values represent changes in levels of support and trust for AI-enabled technologies by treatment conditions compared to the control condition.

We also evaluated the degree to which individuals understand these technologies, recording responses to the question “I have sufficient understanding of AI and how it works across domains.” As Fig 4 below suggests, the relationship is nearly inverted relative to the trust and support dependent variables in Fig 3 above. Compared to the baseline or control group, individuals seem to believe they understand social media content moderation less compared to the control condition of generic AI-enabled technology than for AI-enabled cars, with cars registering more understanding than the control for both enhance (10.14%, *p* < 0.001) and substitute (5.72%, *p* < 0.10) for human judgment. This evidence is consistent with observational data showing that 94% of Americans are aware of efforts to develop autonomous or driverless vehicles [81]. Indeed, approximately 92% of new vehicles have some driver assist function (e.g., automated speed through adaptive cruise control) that is based on AI [82], which may account for the increased understanding of AI-enhanced cars.

Social media content moderation showed the lowest levels of understanding for both enhance (0.86%, *p* < 0.81) and substitution (4.76%, *p* < 0.14) of human judgment. This finding is consistent with previous studies showing that algorithmic content moderation online remains opaque and difficult to audit or understand [83]. Similar to our results above, we also observe that the public seems to believe they understand AI-enabled drones that substitute for human judgment (6.13%, *p* < 0.05) more than other AI-enabled technologies, which may reflect broader societal narratives on the future ubiquity of military robots on the battlefield [84].

Modelling these results in a regression framework, both with and without the incorporation of relevant controls to capture variation across members of the public, also reflects lower levels of support and trust for social media content moderation compared to other AI-enabled technologies (see Supplemental Information for the regression output tables). These results are consistent in both the basic and full regression models and for the use of social media content moderation to enhance and substitute for human judgment. In the full model, for example, social media content moderation to substitute for human judgment results in the lowest degree of support (β = -0.66, *p* < 0.01) and trust (β = -0.64, *p* < 0.01) of any experimental treatment.

To help explain these results, paying special attention to social media content moderation to substitute for human judgment (experimental group four), we then adopted causal mediation analysis. To calculate the proportion of the indirect treatment effect on the outcome explained by our five mediators, we multiplied the effect of the treatment on the mediators by the effect of the mediators on the outcome and then divided by the total treatment effect. This approach replicates a method proposed [85] and adopted by other scholars [64, 86, 87].

**Fig 5:**
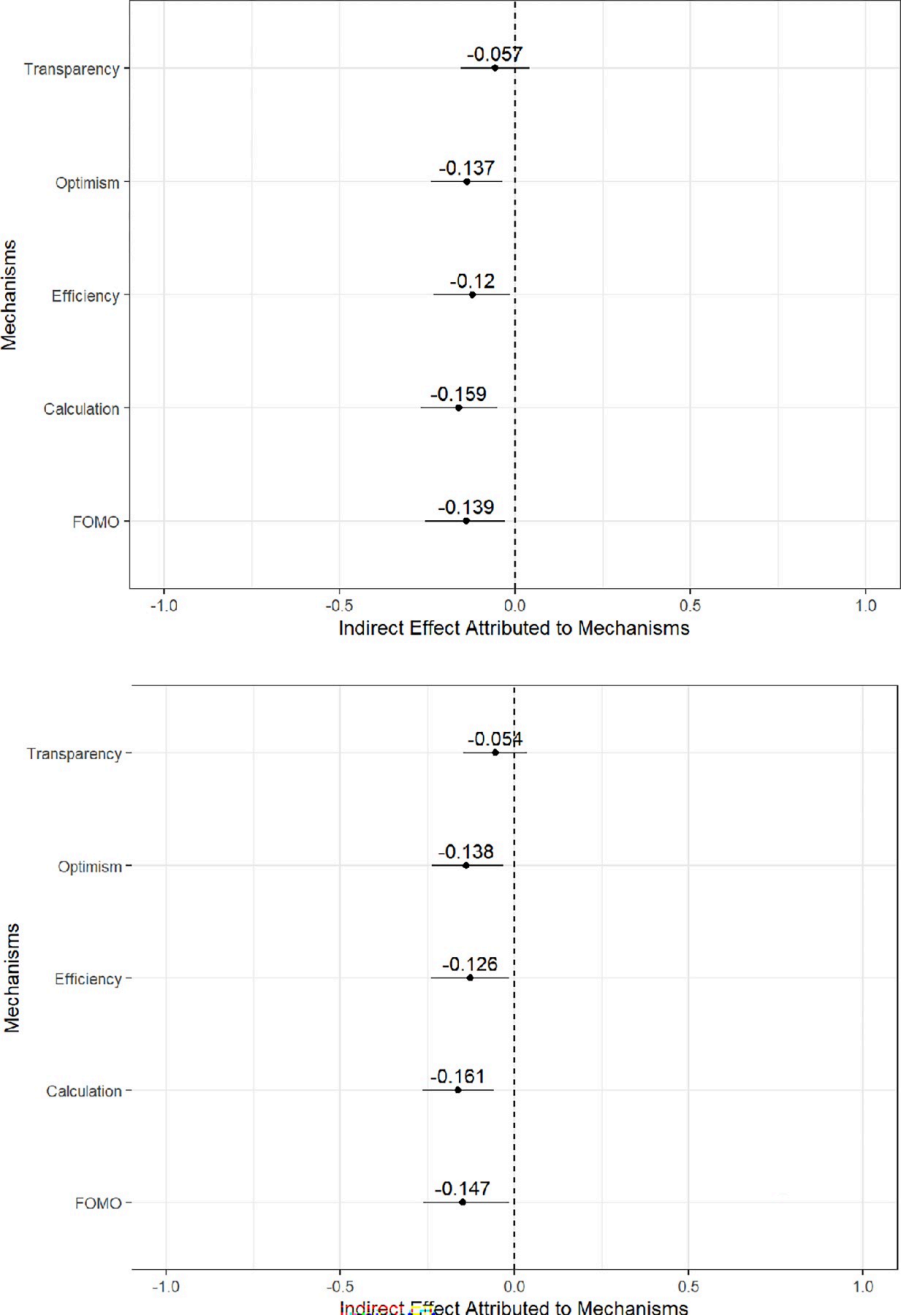
Factors mediating trust in ai-enabled technology. *Caption*: The top graph is for support, and the second graph is for trust. 95% confidence intervals are in whiskers about each point estimate. Whiskers that cross the dashed line at zero denote non-statistically significant results. Some values are negative because the effect of the mechanism acts in the opposite direction of the overall treatment effect.

As indicated in Fig 5 below, we found that those assigned to experimental group four were less likely to say that they believe the appropriate guardrails may not be in place today but they feel confident that those will be in place in the future (13.7%, *p* < 0.01). Similarly, those in experimental group four were 12% (*p* < 0.02) less likely to report that AI was an effective substitute for humans doing the same task, 15.9% (*p* < 0.002) less likely to report that AI might introduce risks but would nonetheless be more likely to generate overall benefits, and 13.9% (*p* < 0.008) less likely to report a fear of missing out. We found that transparency did not exercise a statistically significant shaping effect on overall attitudes of support and trust, indicating that the public emphasizes this consideration less than other factors when determining their use of AI-enabled technologies. Together, then, four of the potential five mediators exercise a *negative* indirect effect on the overall treatment effect, meaning respondents are not generally hopeful about the merits of AI-enabled technologies in terms of social media content moderation that substitutes for human judgment for a variety of reasons.

Though useful, the Baron and Kenney [85] method has no significance test. To assess the strength of the results, we ran a robustness check using the Sobel-Goodman mediation test. In effect, these are T-Tests that show whether the indirect effect of the mediators on the overall treatment effect is statistically different from zero. The Sobel-Goodman mediation tests show significant mediating effects for FOMO (*p* = 0.01), calculation (*p* = 0.003), efficiency (*p* = 0.02), and optimism (*p =* 0.007) in terms of support, and nearly identical results in terms of trust. Consistent with our findings above, the mediating effect for transparency was not statistically significant. Therefore, we can be reasonably assured that four of the five mediators exercise some effect on overall attitudes of support and trust.

## Discussion

To the best of our knowledge, this research is the first study of trust and behavior toward AI-enabled technologies across different use cases and modalities. Previous studies investigate public opinion toward specific technologies, such as autonomous drones and vehicles, or for some AI-applications, including autonomy in the workplace [16, 88, 89]. By contrast, our conjoint-based study offers methodological advancements because it allows us to understand the particular features of the AI-enabled technology that affect attitudes ranging from trust to support for its use, disconnects between the two, and variation on the basis of demographic factors. We then go further to understand the basis of public trust and support, investigating theoretically-grounded mechanisms in a second novel study.

Our analysis shows, first, the existence of a trust paradox, wherein support for the use of AI-enabled technologies is higher than the trust for those same technologies in certain domains, and at certain autonomy and precision levels. The highest level of precision, with fewer mistakes, was considerably more likely to elicit both trust and support. These findings parallel those of public uptake of vaccines, for example, where efficacy is strongly correlated with willingness to receive a vaccine [90].

Perceptions of controllability also play a role, in which willingness to use a particular application increases in a mixed-initiative setting in which humans can override or interact with the machine versus either full autonomy or full human control. Indeed, a recent report published by the Center for Strategic and International Studies echoes this sentiment among U.S. defense experts, stating there are “incredible new opportunities for human-machine teamwork, in which the roles of humans and machines are optimized based upon what each does best” [91]. In light of this potential, the U.S. Air Force’s “loyal wingman” concept enables a pilot of a manned aircraft, such as a F-22 Raptor or F-35 Lightning II, to deploy and maneuver drones in support of mission objectives [92].

Further, the conjoint pointed to a strong preference for AI-enabled technology in the police surveillance domain. Whereas the public was agnostic on most domains, including general surgery, battlefield drones, social media content moderation, and autonomous vehicles, they were substantially more supportive of AI-enabled police surveillance. This tracks with public opinion polls showing a large plurality of adults report that they believe this technology would be beneficial for society [93]. Populations in other countries such as Australia and the United Kingdom have registered greater levels of skepticism, which raises the question about cross-national variation that we suggest should be taken up by future research [94].

In terms of mechanisms, we both theorized about why individuals might trust or support AI-enabled technologies and found that several factors play a role. These include FOMO, belief that the benefits outweigh the risks, support for the view that the technology creates efficient substitutes for tasks that are too dull, dirty, or dangerous for humans, and optimism about the way that safety features are improving to reduce the potential risks imposed by emerging technologies. These attitudes are characterized by a degree of technological optimism that improvements in innovation will provide more sustainable options over time, “an article of faith” according to critics [95]. Further, individual attitudes are broadly consistent with an expected utility calculation that acknowledges the risk that technology poses but expects to derive some form of value from its adoption [96]. While these mediators do not constitute an exhaustive list, they are relevant factors drawn from the literature, and future research could investigate additional mechanisms.

Taken together, our analysis offers both theoretical and empirical insights on public attitudes toward AI-enabled technologies. Beyond the trust paradox, our findings point to variation in support and trust on the basis of domain, precision, and human involvement. Understanding the nature of public concerns and support across a range of applications and modalities is long overdue and this research offers an initial look at how Americans consider AI-enabled technologies. Although it is an important step in understanding public attitudes and behaviors, as well as key factors in societal uptake of or resistance to new technologies [97], future research should consider additional domains, such as AI in the energy sector, manufacturing, communication [98], and politics [99] to understand additional variation depending on the use case. Further, others could introduce the role of bias and a spectrum of consequences to flesh out public tolerance for the range of unintended outcomes of these technologies [100]. The field of AI is rapidly evolving and research on the public uptake and resistance to these technologies will have to evolve alongside those developments.

## Supporting information

S1 Table OLS table (Treatments only). Caption::Conjoint average marginal component effects (AMCE) per attribute level. We use cars, human only autonomy, 85% precision, and community and individual regulations as referents for domain, autonomy, precision, and regulator respectively. The dependent variable is a 5-point Likert scale.
(DOCX)

S2 Table OLS table with controls. Caption: Conjoint average marginal component effects (AMCE) per attribute level. We use cars, human only autonomy, 85% precision, and community and individual regulations as referents for domain, autonomy, precision, and regulator respectively. The dependent variable is a 5-point Likert scale. This model includes additional levels of control variables, specifically income, education, and ethnicity.
(DOCX)

S3 Table OLS table with interaction terms. Note: Conjoint average marginal component (AMCE) effects per attribute level. We use cars, human only autonomy, 85% precision, and community and individual regulations as referents for domain, autonomy, precision, and regulator respectively. The dependent variable is a 5-point Likert scale. Here we interact ethnicity with domain and do not find strong heterogeneous treatment effects.
(DOCX)

S4 Table Conjoint summary statistics. (DOCX)

S5 Table Mediation analysis summary statistics. (DOCX)

S6 Table Support for AI in different domains and for different purposes. (DOCX)

S7 Table Trust for AI in different domains and for different purposes. (DOCX)

S8 Table Understanding for AI in different domains and for different purposes. (DOCX)

S1 File Survey instrument. (DOCX)

S2 File (DOCX)
