# Supplementary material for: Exploring the artificial intelligence “Trust paradox”: Evidence from a survey experiment in the United States
Source: PLoS One. 2023 Jul 18;18(7):e0288109. doi: 10.1371/journal.pone.0288109 (PMC10353804; doi:10.1371/journal.pone.0288109)
Supplement: S1 Table — Caption::Conjoint average marginal component effects (AMCE) per attribute level. We use cars, human only autonomy, 85% precision, and community and individual regulations as referents for domain, autonomy, precision, and regulator respectively. The dependent variable is a 5-point Likert scale. (DOCX) [file pone.0288109.s001.docx]

**S1 Table.  Attributes and public preferences on AI-Enabled Technologies (Treatments Only)**

|  | Support | Trust |
| --- | --- | --- |
| (Intercept) | 3.265*** | 3.096*** |
|  | (0.063) | (0.064) |
| Armed drones | 0.052 | 0.037 |
|  | (0.055) | (0.054) |
| General surgery | 0.067 | 0.079 |
|  | (0.053) | (0.054) |
| Police surveillance | 0.150** | 0.113* |
|  | (0.055) | (0.054) |
| Social media content moderation | -0.002 | 0.019 |
|  | (0.055) | (0.054) |
| Full autonomy and no human oversight | -0.128** | -0.114** |
|  | (0.043) | (0.042) |
| Mixed autonomy (human-in-the-loop) | 0.021 | 0.027 |
|  | (0.041) | (0.041) |
| Maximum precision (correct 99% of the time with 1% false positives) | 0.386*** | 0.366*** |
|  | (0.041) | (0.042) |
| Substantial precision (correct 90% of the time with 10% false positives) | 0.122** | 0.086* |
|  | (0.040) | (0.040) |
| Private industry | 0.016 | 0.002 |
|  | (0.039) | (0.040) |
| Public government agencies | 0.038 | -0.004 |
|  | (0.041) | (0.041) |
| Num.Obs. | 5040 | 5040 |
| R2 | 0.024 | 0.021 |
| R2 Adj. | 0.022 | 0.019 |
| RMSE | 1.16 | 1.16 |
| Std.Errors | by: id | by: id |
| + p < 0.1, * p < 0.05, ** p < 0.01, *** p < 0.001 | | |

Caption: Conjoint average marginal component effects (AMCE) per attribute level. We use cars, human only autonomy, 85% precision, and community and individual regulations as referents for domain, autonomy, precision, and regulator respectively. The dependent variable is a 5-point Likert scale.
