# Supplementary material for: Exploring the artificial intelligence “Trust paradox”: Evidence from a survey experiment in the United States
Source: PLoS One. 2023 Jul 18;18(7):e0288109. doi: 10.1371/journal.pone.0288109 (PMC10353804; doi:10.1371/journal.pone.0288109)
Supplement: S2 Table — Caption: Conjoint average marginal component effects (AMCE) per attribute level. We use cars, human only autonomy, 85% precision, and community and individual regulations as referents for domain, autonomy, precision, and regulator respectively. The dependent variable is a 5-point Likert scale. This model includes additional levels of control variables, specifically income, education, and ethnicity. (DOCX) [file pone.0288109.s002.docx]

**S2 Table.  Attributes and public preferences on AI-Enabled Technologies (with Controls)**

|  | Support (1) | Trust (2) |
| --- | --- | --- |
| (Intercept) | 3.934*** | 3.761*** |
|  | (0.283) | (0.296) |
| Armed drones | 0.054 | 0.038 |
|  | (0.054) | (0.053) |
| General surgery | 0.082 | 0.093+ |
|  | (0.051) | (0.052) |
| Police surveillance | 0.154** | 0.116* |
|  | (0.053) | (0.053) |
| Social media content moderation | -0.011 | 0.014 |
|  | (0.053) | (0.052) |
| Full autonomy and no human oversight | -0.130** | -0.113** |
|  | (0.042) | (0.041) |
| Mixed autonomy | 0.026 | 0.032 |
|  | (0.040) | (0.040) |
| Maximum precision | 0.397*** | 0.378*** |
|  | (0.040) | (0.041) |
| Substantial precision | 0.110** | 0.077* |
|  | (0.039) | (0.039) |
| Private industry | 0.018 | 0.005 |
|  | (0.038) | (0.039) |
| Public government agencies | 0.035 | -0.007 |
|  | (0.041) | (0.041) |
| Male | 0.189*** | 0.216*** |
|  | (0.057) | (0.057) |
| Conservatism | -0.039* | -0.035+ |
|  | (0.018) | (0.018) |
| 10,000 to 24,999 | 0.014 | 0.081 |
|  | (0.120) | (0.120) |
| 25,000 to 49,999 | 0.041 | 0.071 |
|  | (0.108) | (0.109) |
| 50,000 to 74,999 | 0.020 | 0.019 |
|  | (0.120) | (0.121) |
| 75,000 to 99,999 | 0.193 | 0.153 |
|  | (0.130) | (0.133) |
| 100,000+ | 0.181 | 0.172 |
|  | (0.131) | (0.132) |
| High School / GED | -0.392* | -0.438** |
|  | (0.177) | (0.169) |
| Some College | -0.304+ | -0.408* |
|  | (0.177) | (0.169) |
| 2-year College Degree | -0.262 | -0.313+ |
|  | (0.187) | (0.178) |
| 4-year College Degree | -0.158 | -0.255 |
|  | (0.183) | (0.176) |
| Post-Baccalaureate Degree + | -0.031 | -0.054 |
|  | (0.196) | (0.188) |
| Asian | -0.050 | -0.063 |
|  | (0.206) | (0.233) |
| Black | 0.085 | 0.084 |
|  | (0.199) | (0.228) |
| Hispanic/Latino | -0.059 | -0.075 |
|  | (0.207) | (0.231) |
| Native Hawaiian and Other Pacific Islander | 0.156 | 0.164 |
|  | (0.242) | (0.280) |
| White, Non-Hispanic | -0.083 | -0.080 |
|  | (0.189) | (0.216) |
| Age | -0.008*** | -0.008*** |
|  | (0.002) | (0.002) |
| Num.Obs. | 5040 | 5040 |
| R2 | 0.077 | 0.071 |
| R2 Adj. | 0.072 | 0.066 |
| RMSE | 1.12 | 1.13 |
| Std.Errors | by: id | by: id |
| + p < 0.1, * p < 0.05, ** p < 0.01, *** p < 0.001 | | |

Caption: Conjoint average marginal component effects (AMCE) per attribute level. We use cars, human only autonomy, 85% precision, and community and individual regulations as referents for domain, autonomy, precision, and regulator respectively. The dependent variable is a 5-point Likert scale. This model includes additional levels of control variables, specifically income, education, and ethnicity.
