# Supplementary material for: Exploring the artificial intelligence “Trust paradox”: Evidence from a survey experiment in the United States
Source: PLoS One. 2023 Jul 18;18(7):e0288109. doi: 10.1371/journal.pone.0288109 (PMC10353804; doi:10.1371/journal.pone.0288109)
Supplement: S3 Table — Note: Conjoint average marginal component (AMCE) effects per attribute level. We use cars, human only autonomy, 85% precision, and community and individual regulations as referents for domain, autonomy, precision, and regulator respectively. The dependent variable is a 5-point Likert scale. Here we interact ethnicity with domain and do not find strong heterogeneous treatment effects. (DOCX) [file pone.0288109.s003.docx]

| S3 Table: OLS Table with Full Interaction Terms | Support | Trust |
| --- | --- | --- |
| (Intercept) | 3.637*** | 3.595*** |
|  | (0.312) | (0.278) |
| Armed drones | 0.094 | 0.068 |
|  | (0.067) | (0.065) |
| General surgery | 0.138* | 0.134* |
|  | (0.063) | (0.063) |
| Police surveillance | 0.206** | 0.170** |
|  | (0.066) | (0.065) |
| Social media content moderation | 0.012 | 0.055 |
|  | (0.067) | (0.066) |
| Asian | 0.183 | 0.057 |
|  | (0.268) | (0.238) |
| Black | 0.527* | 0.420+ |
|  | (0.254) | (0.228) |
| Hispanic/Latino | 0.353 | 0.168 |
|  | (0.266) | (0.230) |
| Native Hawaiian and Other Pacific Islander | 0.686* | 0.407 |
|  | (0.329) | (0.394) |
| White, Non-Hispanic | 0.171 | 0.050 |
|  | (0.237) | (0.199) |
| Full autonomy and no human oversight | -0.131** | -0.114** |
|  | (0.042) | (0.041) |
| Mixed autonomy (human-in-the-loop) | 0.024 | 0.031 |
|  | (0.041) | (0.041) |
| Maximum precision (correct 99% of the time with 1% false positives) | 0.400*** | 0.379*** |
|  | (0.040) | (0.041) |
| Substantial precision (correct 90% of the time with 10% false positives) | 0.110** | 0.077* |
|  | (0.039) | (0.039) |
| Private industry | 0.019 | 0.006 |
|  | (0.038) | (0.039) |
| Public government agencies | 0.036 | -0.006 |
|  | (0.041) | (0.041) |
| Male | 0.190*** | 0.215*** |
|  | (0.057) | (0.057) |
| Conservatism | -0.039* | -0.035+ |
|  | (0.018) | (0.018) |
| 10,000 to 24,999 | 0.016 | 0.084 |
|  | (0.120) | (0.120) |
| 25,000 to 49,999 | 0.044 | 0.073 |
|  | (0.108) | (0.109) |
| 50,000 to 74,999 | 0.017 | 0.016 |
|  | (0.120) | (0.121) |
| 75,000 to 99,999 | 0.192 | 0.153 |
|  | (0.130) | (0.133) |
| 100,000+ | 0.177 | 0.170 |
|  | (0.131) | (0.132) |
| High School / GED | -0.384* | -0.433** |
|  | (0.176) | (0.167) |
| Some College | -0.301+ | -0.408* |
|  | (0.176) | (0.167) |
| 2-year College Degree | -0.252 | -0.306+ |
|  | (0.186) | (0.177) |
| 4-year College Degree | -0.151 | -0.250 |
|  | (0.181) | (0.174) |
| Post-Baccalaureate Degree + | -0.022 | -0.049 |
|  | (0.195) | (0.186) |
| Age | -0.008*** | -0.008*** |
|  | (0.002) | (0.002) |
| American Indian and Alaskan Native × domain = armed drones | 0.393 | 0.267 |
|  | (0.380) | (0.494) |
| Asian × domain = armed drones | 0.122 | 0.104 |
|  | (0.165) | (0.153) |
| Black × domain = armed drones | -0.203 | -0.242 |
|  | (0.144) | (0.152) |
| Hispanic/Latino × domain = armed drones | -0.268 | -0.103 |
|  | (0.193) | (0.196) |
| Native Hawaiian and Other Pacific Islander × domain = armed drones | -0.631* | -0.222 |
|  | (0.283) | (0.533) |
| American Indian and Alaskan Native × domain = general surgery | 0.052 | -0.039 |
|  | (0.323) | (0.200) |
| Asian × domain = general surgery | -0.143 | -0.129 |
|  | (0.172) | (0.187) |
| Black × domain = general surgery | -0.377* | -0.288+ |
|  | (0.152) | (0.164) |
| Hispanic/Latino × domain = general surgery | 0.079 | 0.100 |
|  | (0.164) | (0.167) |
| Native Hawaiian and Other Pacific Islander × domain = general surgery | -0.188 | -0.127 |
|  | (0.258) | (0.470) |
| American Indian and Alaskan Native × domain = police surveillance | 0.441 | 0.051 |
|  | (0.300) | (0.374) |
| Asian × domain = police surveillance | -0.122 | 0.048 |
|  | (0.196) | (0.192) |
| Black × domain = police surveillance | -0.189 | -0.255 |
|  | (0.147) | (0.159) |
| Hispanic/Latino × domain = police surveillance | -0.357* | -0.328* |
|  | (0.169) | (0.163) |
| Native Hawaiian and Other Pacific Islander × domain = police surveillance | 0.825* | 0.778 |
|  | (0.415) | (0.759) |
| American Indian and Alaskan Native × domain = social media content moderation | 0.433 | 0.379 |
|  | (0.313) | (0.274) |
| Asian × domain = social media content moderation | 0.226 | 0.044 |
|  | (0.194) | (0.161) |
| Black × domain = social media content moderation | -0.160 | -0.225 |
|  | (0.141) | (0.149) |
| Hispanic/Latino × domain = social media content moderation | -0.210 | -0.211 |
|  | (0.160) | (0.155) |
| Native Hawaiian and Other Pacific Islander × domain = social media content moderation | -0.522 | -0.315 |
|  | (0.581) | (0.634) |
| Num.Obs. | 5040 | 5040 |
| R2 | 0.081 | 0.074 |
| R2 Adj. | 0.073 | 0.065 |
| RMSE | 1.12 | 1.13 |
| Std.Errors | by: id | by: id |
| + p < 0.1, * p < 0.05, ** p < 0.01, *** p < 0.001 | | |

Caption: Conjoint average marginal component (AMCE) effects per attribute level. We use cars, human only autonomy, 85% precision, and community and individual regulations as referents for domain, autonomy, precision, and regulator respectively. The dependent variable is a 5-point Likert scale. Here we interact ethnicity with domain and do not find strong heterogeneous treatment effects.
