# Supplementary material for: Exploring the artificial intelligence “Trust paradox”: Evidence from a survey experiment in the United States
Source: PLoS One. 2023 Jul 18;18(7):e0288109. doi: 10.1371/journal.pone.0288109 (PMC10353804; doi:10.1371/journal.pone.0288109)
Supplement: S4 Table — (DOCX) [file pone.0288109.s004.docx]

S4 Table: Conjoint Summary Statistics

|  |  | N | % |
| --- | --- | --- | --- |
| Ideology | Extremely Conservative | 107 | 10.62 |
|  | Conservative | 124 | 12.30 |
|  | Slightly Conservative | 94 | 9.33 |
|  | Moderate/Unsure | 361 | 35.81 |
|  | Slightly Liberal | 96 | 9.52 |
|  | Liberal | 125 | 12.40 |
|  | Extremely Liberal | 101 | 10.02 |
| Gender | Male | 494 | 49.01 |
|  | Female/Other | 514 | 50.99 |
| Income | l.t. 10,000 | 92 | 9.13 |
|  | 10,000 to 24,999 | 154 | 15.28 |
|  | 25,000 to 49,999 | 291 | 28.87 |
|  | 50,000 to 74,999 | 203 | 20.14 |
|  | 75,000 to 99,999 | 104 | 10.32 |
|  | 100,000+ | 164 | 16.27 |
| Ethnicity | American Indian and Alaskan Native | 12 | 1.19 |
|  | Asian | 55 | 5.46 |
|  | Black | 132 | 13.10 |
|  | Hispanic/Latino | 80 | 7.94 |
|  | Native Hawaiian and Other Pacific Islander | 5 | 0.50 |
|  | White, Non-Hispanic | 724 | 71.83 |
| Education | l.t. HS | 24 | 2.38 |
|  | High School / GED | 264 | 26.19 |
|  | Some College | 246 | 24.40 |
|  | 2-year College Degree | 127 | 12.60 |
|  | 4-year College Degree | 220 | 21.83 |
|  | Post-Baccalaureate Degree + | 127 | 12.60 |
| Age | 18-19 | 20 | 1.98 |
|  | 20-34 | 302 | 29.96 |
|  | 35-44 | 186 | 18.45 |
|  | 45-54 | 200 | 19.84 |
|  | 55-64 | 130 | 12.90 |
|  | 65+ | 170 | 16.87 |

Total *N* = 1,008.
