# Supplementary material for: Exploring the artificial intelligence “Trust paradox”: Evidence from a survey experiment in the United States
Source: PLoS One. 2023 Jul 18;18(7):e0288109. doi: 10.1371/journal.pone.0288109 (PMC10353804; doi:10.1371/journal.pone.0288109)
Supplement: S5 Table — (DOCX) [file pone.0288109.s005.docx]

S5 Table: Mediation Analysis Summary Statistics

| **Group** | **Overall**, N = 1,002 | **Control**, N = 147 | **T1**, N = 136 | **T2**, N = 147 | **T3**, N = 144 | **T4**, N = 142 | **T5**, N = 141 | **T6**, N = 145 |
| --- | --- | --- | --- | --- | --- | --- | --- | --- |
| **Sex** |  |  |  |  |  |  |  |  |
| Men | 492 (49.1%) | 55 (37.4%) | 72 (52.9%) | 75 (51.0%) | 82 (56.9%) | 67 (47.2%) | 70 (49.6%) | 71 (49.0%) |
| Women | 510 (50.9%) | 92 (62.6%) | 64 (47.1%) | 72 (49.0%) | 62 (43.1%) | 75 (52.8%) | 71 (50.4%) | 74 (51.0%) |
| **Age** |  |  |  |  |  |  |  |  |
| 19-24 | 132 (13.2%) | 25 (17.0%) | 15 (11.0%) | 19 (12.9%) | 17 (11.8%) | 16 (11.3%) | 17 (12.1%) | 23 (15.9%) |
| 26-35 | 214 (21.4%) | 32 (21.8%) | 32 (23.5%) | 36 (24.5%) | 26 (18.1%) | 26 (18.3%) | 36 (25.5%) | 26 (17.9%) |
| 36-45 | 189 (18.9%) | 20 (13.6%) | 26 (19.1%) | 24 (16.3%) | 32 (22.2%) | 28 (19.7%) | 28 (19.9%) | 31 (21.4%) |
| 46-55 | 187 (18.7%) | 31 (21.1%) | 27 (19.9%) | 31 (21.1%) | 24 (16.7%) | 31 (21.8%) | 19 (13.5%) | 24 (16.6%) |
| 56-65 | 126 (12.6%) | 22 (15.0%) | 18 (13.2%) | 19 (12.9%) | 16 (11.1%) | 16 (11.3%) | 15 (10.6%) | 20 (13.8%) |
| Over 66 | 154 (15.4%) | 17 (11.6%) | 18 (13.2%) | 18 (12.2%) | 29 (20.1%) | 25 (17.6%) | 26 (18.4%) | 21 (14.5%) |
| **Ethnicity** |  |  |  |  |  |  |  |  |
| American Indian, Alaskan Native | 12 (1.2%) | 4 (2.7%) | 1 (0.7%) | 0 (0.0%) | 1 (0.7%) | 4 (2.8%) | 0 (0.0%) | 2 (1.4%) |
| Asian | 56 (5.6%) | 7 (4.8%) | 4 (2.9%) | 12 (8.2%) | 2 (1.4%) | 12 (8.5%) | 10 (7.1%) | 9 (6.2%) |
| Black | 131 (13.1%) | 23 (15.6%) | 20 (14.7%) | 16 (10.9%) | 17 (11.8%) | 19 (13.4%) | 21 (14.9%) | 15 (10.3%) |
| Hispanic | 79 (7.9%) | 15 (10.2%) | 11 (8.1%) | 6 (4.1%) | 14 (9.7%) | 14 (9.9%) | 6 (4.3%) | 13 (9.0%) |
| Native Hawaiian, Other Pacific Islander | 5 (0.5%) | 0 (0.0%) | 2 (1.5%) | 0 (0.0%) | 0 (0.0%) | 2 (1.4%) | 0 (0.0%) | 1 (0.7%) |
| White, Non-Hispanic | 719 (71.8%) | 98 (66.7%) | 98 (72.1%) | 113 (76.9%) | 110 (76.4%) | 91 (64.1%) | 104 (73.8%) | 105 (72.4%) |
| **Education** |  |  |  |  |  |  |  |  |
| No High School | 24 (2.4%) | 3 (2.0%) | 3 (2.2%) | 8 (5.4%) | 1 (0.7%) | 4 (2.8%) | 3 (2.1%) | 2 (1.4%) |
| High School | 260 (25.9%) | 45 (30.6%) | 37 (27.2%) | 46 (31.3%) | 27 (18.8%) | 32 (22.5%) | 29 (20.6%) | 44 (30.3%) |
| Some College | 243 (24.3%) | 33 (22.4%) | 34 (25.0%) | 31 (21.1%) | 46 (31.9%) | 30 (21.1%) | 41 (29.1%) | 28 (19.3%) |
| 2-Year Degree | 127 (12.7%) | 15 (10.2%) | 18 (13.2%) | 16 (10.9%) | 15 (10.4%) | 21 (14.8%) | 21 (14.9%) | 21 (14.5%) |
| 4-Year Degree | 220 (22.0%) | 32 (21.8%) | 30 (22.1%) | 26 (17.7%) | 39 (27.1%) | 35 (24.6%) | 27 (19.1%) | 31 (21.4%) |
| Advanced Degree | 128 (12.8%) | 19 (12.9%) | 14 (10.3%) | 20 (13.6%) | 16 (11.1%) | 20 (14.1%) | 20 (14.2%) | 19 (13.1%) |
| **Income** |  |  |  |  |  |  |  |  |
| < $10,000 | 91 (9.1%) | 16 (10.9%) | 9 (6.6%) | 17 (11.6%) | 7 (4.9%) | 14 (9.9%) | 16 (11.3%) | 12 (8.3%) |
| $10,000-$24,999 | 153 (15.3%) | 25 (17.0%) | 17 (12.5%) | 16 (10.9%) | 26 (18.1%) | 26 (18.3%) | 23 (16.3%) | 20 (13.8%) |
| $25,000-$49,999 | 287 (28.6%) | 43 (29.3%) | 45 (33.1%) | 42 (28.6%) | 36 (25.0%) | 40 (28.2%) | 35 (24.8%) | 46 (31.7%) |
| $50,000-$74,999 | 203 (20.3%) | 24 (16.3%) | 32 (23.5%) | 34 (23.1%) | 30 (20.8%) | 23 (16.2%) | 34 (24.1%) | 26 (17.9%) |
| $75,000-$99,999 | 104 (10.4%) | 16 (10.9%) | 13 (9.6%) | 18 (12.2%) | 16 (11.1%) | 16 (11.3%) | 11 (7.8%) | 14 (9.7%) |
| > $100,000 | 164 (16.4%) | 23 (15.6%) | 20 (14.7%) | 20 (13.6%) | 29 (20.1%) | 23 (16.2%) | 22 (15.6%) | 27 (18.6%) |
