# Supplementary material for: Exploring the artificial intelligence “Trust paradox”: Evidence from a survey experiment in the United States
Source: PLoS One. 2023 Jul 18;18(7):e0288109. doi: 10.1371/journal.pone.0288109 (PMC10353804; doi:10.1371/journal.pone.0288109)
Supplement: S6 Table — (DOCX) [file pone.0288109.s006.docx]

S6 Table: Support for AI in Different Domains and for Different Purposes

| **Support for AI in Different Domains and for Different Purposes** | | | | | |
| --- | --- | --- | --- | --- | --- |
|  | | | | | |
|  | Support | | | | |
|  | (1) | (2) | (3) | (4) | (5) |
|  | | | | | |
| T1 (Cars, Enhance) | -0.30^**^ | -0.32^**^ | -0.37^***^ | -0.38^***^ | -0.39^***^ |
|  | (-0.56, -0.04) | (-0.57, -0.07) | (-0.62, -0.12) | (-0.63, -0.13) | (-0.64, -0.14) |
|  |  |  |  |  |  |
| T2 (Cars, Substitute) | -0.44^***^ | -0.45^***^ | -0.50^***^ | -0.49^***^ | -0.48^***^ |
|  | (-0.70, -0.19) | (-0.70, -0.20) | (-0.74, -0.25) | (-0.74, -0.25) | (-0.73, -0.24) |
|  |  |  |  |  |  |
| T3 (Online, Enhance) | -0.32^**^ | -0.34^***^ | -0.39^***^ | -0.39^***^ | -0.39^***^ |
|  | (-0.57, -0.06) | (-0.59, -0.09) | (-0.64, -0.14) | (-0.64, -0.14) | (-0.64, -0.15) |
|  |  |  |  |  |  |
| TS (Online, Substitute) | -0.61^***^ | -0.62^***^ | -0.66^***^ | -0.66^***^ | -0.66^***^ |
|  | (-0.86, -0.35) | (-0.87, -0.37) | (-0.91, -0.41) | (-0.91, -0.41) | (-0.91, -0.41) |
|  |  |  |  |  |  |
| T5 (Drones, Enhance) | -0.44^***^ | -0.45^***^ | -0.48^***^ | -0.49^***^ | -0.48^***^ |
|  | (-0.69, -0.18) | (-0.70, -0.20) | (-0.73, -0.24) | (-0.74, -0.25) | (-0.73, -0.24) |
|  |  |  |  |  |  |
| T6 (Drones, Substitute) | -0.39^***^ | -0.41^***^ | -0.45^***^ | -0.45^***^ | -0.43^***^ |
|  | (-0.64, -0.13) | (-0.66, -0.16) | (-0.70, -0.20) | (-0.69, -0.20) | (-0.67, -0.18) |
|  |  |  |  |  |  |
| Sex |  | -0.19^***^ | -0.19^***^ | -0.19^***^ | -0.13^*^ |
|  |  | (-0.32, -0.05) | (-0.33, -0.06) | (-0.33, -0.06) | (-0.27, 0.01) |
|  |  |  |  |  |  |
| Age |  | -0.11^***^ | -0.11^***^ | -0.11^***^ | -0.12^***^ |
|  |  | (-0.15, -0.07) | (-0.15, -0.07) | (-0.15, -0.07) | (-0.16, -0.07) |
|  |  |  |  |  |  |
| Education |  | 0.08^***^ | 0.07^**^ | 0.06^**^ | 0.05^*^ |
|  |  | (0.03, 0.13) | (0.01, 0.12) | (0.01, 0.11) | (-0.003, 0.10) |
|  |  |  |  |  |  |
| Race |  | -0.04 | -0.03 | -0.03 | -0.02 |
|  |  | (-0.09, 0.01) | (-0.08, 0.02) | (-0.08, 0.02) | (-0.07, 0.03) |
|  |  |  |  |  |  |
| Income |  | 0.05^**^ | 0.05^**^ | 0.06^**^ | 0.05^**^ |
|  |  | (0.002, 0.10) | (0.002, 0.10) | (0.01, 0.11) | (0.005, 0.10) |
|  |  |  |  |  |  |
| Political Party |  |  | -0.14^***^ | -0.10^**^ | -0.10^**^ |
|  |  |  | (-0.21, -0.08) | (-0.17, -0.02) | (-0.17, -0.02) |
|  |  |  |  |  |  |
| Political Ideology |  |  |  | -0.05^**^ | -0.05^**^ |
|  |  |  |  | (-0.09, -0.01) | (-0.09, -0.01) |
|  |  |  |  |  |  |
| Military Service |  |  |  |  | -0.35^***^ |
|  |  |  |  |  | (-0.54, -0.15) |
|  |  |  |  |  |  |
| Constant | 3.89^***^ | 4.41^***^ | 4.73^***^ | 4.86^***^ | 5.44^***^ |
|  | (3.71, 4.07) | (3.96, 4.85) | (4.27, 5.20) | (4.39, 5.34) | (4.87, 6.01) |
|  |  |  |  |  |  |
| *N* | 1,007 | 1,007 | 1,007 | 1,007 | 1,007 |
| Adjusted R^2^ | 0.02 | 0.08 | 0.09 | 0.10 | 0.11 |
| F Statistic | 4.09^***^ | 8.69^***^ | 9.59^***^ | 9.41^***^ | 9.71^***^ |
|  | | | | | |
| *Notes:* | ^***^Significant at the 1 percent level. | | | | |
|  | ^**^Significant at the 5 percent level. | | | | |
|  | ^*^Significant at the 10 percent level. | | | | |
