# Supplementary material for: Exploring the artificial intelligence “Trust paradox”: Evidence from a survey experiment in the United States
Source: PLoS One. 2023 Jul 18;18(7):e0288109. doi: 10.1371/journal.pone.0288109 (PMC10353804; doi:10.1371/journal.pone.0288109)
Supplement: S7 Table — (DOCX) [file pone.0288109.s007.docx]

S7 Table: Trust for AI in Different Domains and for Different Purposes

| **Trust for AI in Different Domains and for Different Purposes** | | | | | |
| --- | --- | --- | --- | --- | --- |
|  | | | | | |
|  | Trust | | | | |
|  | (1) | (2) | (3) | (4) | (5) |
|  | | | | | |
| T1 (Cars, Enhance) | -0.35^**^ | -0.36^***^ | -0.42^***^ | -0.43^***^ | -0.44^***^ |
|  | (-0.61, -0.08) | (-0.62, -0.11) | (-0.67, -0.16) | (-0.68, -0.17) | (-0.69, -0.18) |
|  |  |  |  |  |  |
| T2 (Cars, Substitute) | -0.47^***^ | -0.47^***^ | -0.52^***^ | -0.52^***^ | -0.50^***^ |
|  | (-0.73, -0.21) | (-0.73, -0.22) | (-0.77, -0.27) | (-0.77, -0.26) | (-0.76, -0.25) |
|  |  |  |  |  |  |
| T3 (Online, Enhance) | -0.26^*^ | -0.28^**^ | -0.33^**^ | -0.33^**^ | -0.33^**^ |
|  | (-0.52, 0.002) | (-0.54, -0.02) | (-0.59, -0.07) | (-0.59, -0.08) | (-0.59, -0.08) |
|  |  |  |  |  |  |
| TS (Online, Substitute) | -0.59^***^ | -0.60^***^ | -0.64^***^ | -0.64^***^ | -0.64^***^ |
|  | (-0.85, -0.32) | (-0.86, -0.35) | (-0.89, -0.38) | (-0.90, -0.39) | (-0.89, -0.39) |
|  |  |  |  |  |  |
| T5 (Drones, Enhance) | -0.38^***^ | -0.39^***^ | -0.43^***^ | -0.44^***^ | -0.43^***^ |
|  | (-0.64, -0.12) | (-0.65, -0.14) | (-0.68, -0.17) | (-0.69, -0.18) | (-0.68, -0.17) |
|  |  |  |  |  |  |
| T6 (Drones, Substitute) | -0.31^**^ | -0.33^**^ | -0.37^***^ | -0.37^***^ | -0.35^***^ |
|  | (-0.57, -0.05) | (-0.58, -0.08) | (-0.62, -0.12) | (-0.62, -0.11) | (-0.60, -0.10) |
|  |  |  |  |  |  |
| Sex |  | -0.22^***^ | -0.23^***^ | -0.23^***^ | -0.16^**^ |
|  |  | (-0.36, -0.08) | (-0.37, -0.09) | (-0.37, -0.09) | (-0.31, -0.02) |
|  |  |  |  |  |  |
| Age |  | -0.11^***^ | -0.11^***^ | -0.11^***^ | -0.11^***^ |
|  |  | (-0.15, -0.06) | (-0.15, -0.07) | (-0.15, -0.06) | (-0.16, -0.07) |
|  |  |  |  |  |  |
| Education |  | 0.06^**^ | 0.05^*^ | 0.04 | 0.03 |
|  |  | (0.01, 0.12) | (-0.002, 0.10) | (-0.01, 0.10) | (-0.02, 0.09) |
|  |  |  |  |  |  |
| Race |  | -0.06^**^ | -0.04^*^ | -0.04^*^ | -0.04 |
|  |  | (-0.11, -0.01) | (-0.09, 0.01) | (-0.10, 0.01) | (-0.09, 0.01) |
|  |  |  |  |  |  |
| Income |  | 0.05^*^ | 0.05^*^ | 0.05^*^ | 0.05^*^ |
|  |  | (-0.005, 0.10) | (-0.01, 0.10) | (-0.001, 0.10) | (-0.002, 0.10) |
|  |  |  |  |  |  |
| Political Party |  |  | -0.14^***^ | -0.10^**^ | -0.10^**^ |
|  |  |  | (-0.21, -0.07) | (-0.17, -0.02) | (-0.17, -0.02) |
|  |  |  |  |  |  |
| Political Ideology |  |  |  | -0.05^**^ | -0.05^**^ |
|  |  |  |  | (-0.09, -0.01) | (-0.09, -0.01) |
|  |  |  |  |  |  |
| Military Service |  |  |  |  | -0.37^***^ |
|  |  |  |  |  | (-0.57, -0.17) |
|  |  |  |  |  |  |
| Constant | 3.71^***^ | 4.43^***^ | 4.75^***^ | 4.87^***^ | 5.49^***^ |
|  | (3.53, 3.89) | (3.98, 4.89) | (4.27, 5.23) | (4.39, 5.36) | (4.90, 6.08) |
|  |  |  |  |  |  |
| *N* | 1,007 | 1,007 | 1,007 | 1,007 | 1,007 |
| Adjusted R^2^ | 0.02 | 0.07 | 0.09 | 0.09 | 0.10 |
| F Statistic | 3.76^***^ | 8.12^***^ | 8.94^***^ | 8.73^***^ | 9.14^***^ |
|  | | | | | |
| *Notes:* | ^***^Significant at the 1 percent level. | | | | |
|  | ^**^Significant at the 5 percent level. | | | | |
|  | ^*^Significant at the 10 percent level. | | | | |
