# Supplementary material for: Exploring the artificial intelligence “Trust paradox”: Evidence from a survey experiment in the United States
Source: PLoS One. 2023 Jul 18;18(7):e0288109. doi: 10.1371/journal.pone.0288109 (PMC10353804; doi:10.1371/journal.pone.0288109)
Supplement: S8 Table — (DOCX) [file pone.0288109.s008.docx]

S8 Table: Understanding for AI in Different Domains and for Different Purposes

| **Understanding for AI in Different Domains and for Different Purposes** | | | | | |
| --- | --- | --- | --- | --- | --- |
|  | | | | | |
|  | Understanding | | | | |
|  | (1) | (2) | (3) | (4) | (5) |
|  | | | | | |
| T1 (Cars, Enhance) | 0.39^***^ | 0.36^***^ | 0.33^***^ | 0.32^***^ | 0.31^***^ |
|  | (0.15, 0.63) | (0.13, 0.59) | (0.10, 0.55) | (0.09, 0.55) | (0.08, 0.53) |
|  |  |  |  |  |  |
| T2 (Cars, Substitute) | 0.21^*^ | 0.18 | 0.15 | 0.15 | 0.17 |
|  | (-0.02, 0.44) | (-0.04, 0.41) | (-0.07, 0.38) | (-0.07, 0.38) | (-0.05, 0.39) |
|  |  |  |  |  |  |
| T3 (Online, Enhance) | 0.03 | -0.005 | -0.03 | -0.04 | -0.04 |
|  | (-0.21, 0.27) | (-0.23, 0.22) | (-0.26, 0.19) | (-0.26, 0.19) | (-0.26, 0.19) |
|  |  |  |  |  |  |
| TS (Online, Substitute) | 0.17 | 0.17 | 0.15 | 0.15 | 0.15 |
|  | (-0.06, 0.41) | (-0.05, 0.40) | (-0.08, 0.38) | (-0.08, 0.37) | (-0.07, 0.37) |
|  |  |  |  |  |  |
| T5 (Drones, Enhance) | 0.09 | 0.07 | 0.05 | 0.05 | 0.06 |
|  | (-0.14, 0.33) | (-0.15, 0.30) | (-0.17, 0.28) | (-0.18, 0.28) | (-0.16, 0.29) |
|  |  |  |  |  |  |
| T6 (Drones, Substitute) | 0.23^*^ | 0.20^*^ | 0.17 | 0.17 | 0.20^*^ |
|  | (-0.01, 0.46) | (-0.03, 0.42) | (-0.05, 0.40) | (-0.05, 0.40) | (-0.03, 0.42) |
|  |  |  |  |  |  |
| Sex |  | -0.25^***^ | -0.26^***^ | -0.26^***^ | -0.18^***^ |
|  |  | (-0.38, -0.13) | (-0.38, -0.13) | (-0.38, -0.13) | (-0.30, -0.05) |
|  |  |  |  |  |  |
| Age |  | -0.14^***^ | -0.15^***^ | -0.14^***^ | -0.15^***^ |
|  |  | (-0.18, -0.11) | (-0.18, -0.11) | (-0.18, -0.11) | (-0.19, -0.12) |
|  |  |  |  |  |  |
| Education |  | 0.03 | 0.03 | 0.02 | 0.01 |
|  |  | (-0.01, 0.08) | (-0.02, 0.08) | (-0.02, 0.07) | (-0.04, 0.06) |
|  |  |  |  |  |  |
| Race |  | -0.01 | 0.003 | 0.003 | 0.01 |
|  |  | (-0.05, 0.04) | (-0.04, 0.05) | (-0.04, 0.05) | (-0.03, 0.06) |
|  |  |  |  |  |  |
| Income |  | 0.07^***^ | 0.07^***^ | 0.07^***^ | 0.07^***^ |
|  |  | (0.02, 0.11) | (0.02, 0.11) | (0.02, 0.11) | (0.02, 0.11) |
|  |  |  |  |  |  |
| Political Party |  |  | -0.09^***^ | -0.07^*^ | -0.07^**^ |
|  |  |  | (-0.15, -0.03) | (-0.14, 0.0001) | (-0.14, -0.001) |
|  |  |  |  |  |  |
| Political Ideology |  |  |  | -0.02 | -0.02 |
|  |  |  |  | (-0.06, 0.02) | (-0.05, 0.02) |
|  |  |  |  |  |  |
| Military Service |  |  |  |  | -0.45^***^ |
|  |  |  |  |  | (-0.63, -0.28) |
|  |  |  |  |  |  |
| Constant | 3.47^***^ | 4.17^***^ | 4.37^***^ | 4.42^***^ | 5.17^***^ |
|  | (3.30, 3.63) | (3.77, 4.58) | (3.94, 4.79) | (3.98, 4.85) | (4.65, 5.69) |
|  |  |  |  |  |  |
| *N* | 1,007 | 1,007 | 1,007 | 1,007 | 1,007 |
| Adjusted R^2^ | 0.01 | 0.10 | 0.10 | 0.10 | 0.12 |
| F Statistic | 2.41^**^ | 10.81^***^ | 10.62^***^ | 9.89^***^ | 11.22^***^ |
|  | | | | | |
| *Notes:* | ^***^Significant at the 1 percent level. | | | | |
|  | ^**^Significant at the 5 percent level. | | | | |
|  | ^*^Significant at the 10 percent level. | | | | |
