# Supplementary material for: Exploring the artificial intelligence “Trust paradox”: Evidence from a survey experiment in the United States
Source: PLoS One. 2023 Jul 18;18(7):e0288109. doi: 10.1371/journal.pone.0288109 (PMC10353804; doi:10.1371/journal.pone.0288109)
Supplement: S1 File — (DOCX) [file pone.0288109.s009.docx]

S9 Exhibit: Survey Instrument

(Exempt from the Institutional Review Board for Human Subject Research - IRB Protocol #2004009569)

**1. Informed Participant Consent (Electronic):**

**What the study is about:**

The purpose of this research is to better understand public perceptions of Artificial Intelligence.

**What we will ask you to do:**

We will first ask you a series of background questions. These will include questions about your age, gender, race, income, and education. At no point will you be asked any question which could personally identify you. Thus, we assure you that your responses will be anonymous and confidential. After these, we will ask some brief responses on questions related to Artificial Intelligence. We estimate that the survey will take you about 10 minutes to complete.

**Risks and discomforts:**

We do not anticipate any risks from participating in this research.

**Benefits:**

There are no direct benefits to participating in the survey. Participants may gain some indirect benefits from greater engagement with important political and societal issues. Information from this study may benefit other people now or in the future by helping us better understand what and how Americans think about important policy issues.

**Privacy, confidentiality, and data security:**

All survey data is collected through an anonymous link from the survey research firm Qualtrics. No identifying information, including IP address or physical location, will be collected. We anticipate that your participation in this survey presents no greater risk than everyday use of the Internet.

**Sharing de-identified data collected in this research:**

De-identified data from this study may be shared with the research community at large to advance science and health. To protect your privacy, we do not collect any personal information that could identify you. This is done to ensure that, by current scientific standards and known methods, no one will be able to identify you from the information we share.

**Taking part is voluntary:**

Your participation in this research is voluntary. You have the right to withdraw at any point during the study, for any reason and the data you have submitted to that point will be destroyed and not stored for use.

**If you have questions:**

The main researcher conducting this study is Professor Sarah Kreps at Cornell University. You are encouraged to ask the researcher questions at any time about the nature of the study and the methods that are being used. You may contact them at pal243@cornell.edu. If you have any questions or concerns regarding your rights as a subject in this study, you may contact the Institutional Review Board (IRB) for Human Participants at 607-255-5138 or access their website at http://www.irb.cornell.edu. You may also report your concerns or complaints anonymously through EthicsPoint online at www.hotline.cornell.edu or by calling toll free at 1-866-293-3077. EthicsPoint is an independent organization that serves as a liaison between the University and the person bringing the complaint so that anonymity can be ensured.

**Please indicate below that you agree to participate in this survey:**

- I agree to participate (1)
- I do not agree to participate (2)

**2. Demographic variables:**

1. What is your sex? (Q1)

- Male (1)
- Female (2)
- Other (3)

1. How old are you? (Q2)

- Under 18 (1)
- 19-25 (2)
- 26-35 (3)
- 35-45 (4)
- 46-55 (5)
- 56-65 (6)
- Over 66 (7)

1. What racial or ethnic group best describes you? (Q3)

- American Indian and Alaskan Native (1)
- Asian (2)
- Black (3)
- Hispanic (4)
- Native Hawaiian and Other Pacific Islander (5)
- White, Non-Hispanic (6)

1. What is the highest level of education that you have completed? (Q4)

- Less than high school (1)
- High school (diploma or GED) (2)
- Some college, but no degree (3)
- 2-year college degree (4)
- 4-year college degree (5)
- Advanced or professional degree (MA, MBA, MD, JD, PhD, etc.) (6)

**3. Experiment one:**

*Programming Note: General prompt provided to all respondents.*

Recent advances in Artificial-Intelligence (AI) have enabled the development of new technologies, adopted in many industries. In the following section, you’ll be presented with hypothetical applications of these AI-enabled technologies and asked about your thoughts.

a. Do you agree to read the details very carefully and then give your most thoughtful answers? (Q5)

- Yes (1)
- No (2)

*Programming Note: Treatment scenarios are randomized.*

**Scenario Treatments (Domain, Autonomy, Regulation, Precision):** Consider the use of AI in **DOMAIN** with **AUTONOMY**. The technology is regulated by **REGULATION** and has **PRECISION** in this setting.

| **ATTRIBUTES** | **LEVELS** |
| --- | --- |
| Domain | - Cars  - Armed drones  - General surgery  - Social media content moderation  - Police surveillance |
| Degree of Autonomy | - Fully autonomous (no human in the loop)  - Mixed-initiative (on-/off switch between human and machine)  - Manual (no autonomy) |
| Regulation | - Public government agencies  - Private industry  - Individual or community users |
| Precision | - Maximum precision—a model that produces very few false positives, and is correct 99% of the time.  - Substantial precision—a model that produces 10% false positives, or in other words, is correct 90% of the time.  - Moderate precision—a model that produces 15% false positives, or in other words, is correct 85% of the time. |

**4. Adjudicating support and trust:**

*Programming Note: As per our 5x3x3x3 conjoint survey design, rendering 135 potential AI use cases, respondents will view one hypothetical scenario and then answer several questions*.

1. Do you ***support*** the use of AI under these circumstances? (conjoint_dv_1)

- Strongly Agree (5)
- Agree (4)
- Neither Agree Nor Disagree (3)
- Disagree (2)
- Strongly Disagree (1)

1. Do you ***trust*** the use of AI under these circumstances? (conjoint_dv_2)

- Strongly Agree (5)
- Agree (4)
- Neither Agree Nor Disagree (3)
- Disagree (2)
- Strongly Disagree (1)

**5. Experiment two:**

*Programming Note: General prompt provided to all respondents.*

The following scenario describes a technology enabled by Artificial-Intelligence (AI). I will describe the circumstances and ask you questions about your thoughts.

a. Do you agree to read the details very carefully and then give your most thoughtful answers? (Q6)

- Yes (1)
- No (2)

*Programming Note: Treatment scenarios are randomized.*

**Scenario #1 Treatment (Self-Driving Cars, Enhance Human Judgment):** “In recent years, advancements in Artificial-Intelligence (AI) have led to the emergence of new technologies. One notable AI-enabled technology is **self-driving cars**. The goal of this AI-enabled technology is to **enhance human judgment** in this setting.”

**Scenario #2 Treatment (Self-Driving Cars, Substitute for Human Judgment):** “In recent years, advancements in Artificial-Intelligence (AI) have led to the emergence of new technologies. One notable AI-enabled technology is **self-driving cars**. The goal of this AI-enabled technology is to **substitute for human judgment** in this setting.”

**Scenario #3 Treatment (Social Media Content Moderation, Enhance Human Judgment):** “In recent years, advancements in Artificial-Intelligence (AI) have led to the emergence of new technologies. One notable AI-enabled technology is **social media content moderation**. The goal of this AI-enabled technology is to **enhance human judgment** in this setting.”

**Scenario #4 Treatment (Social Media Content Moderation, Substitute for Human Judgment):** “In recent years, advancements in Artificial-Intelligence (AI) have led to the emergence of new technologies. One notable AI-enabled technology is **online content moderation**. The goal of this AI-enabled technology is to **substitute for human judgment** in this setting.”

**Scenario #5 Treatment (Armed Drones, Enhance Human Judgment):** “In recent years, advancements in Artificial-Intelligence (AI) have led to the emergence of new technologies. One notable AI-enabled technology is **armed drones**. The goal of this AI-enabled technology is to **enhance human judgment** in this setting.”

**Scenario #6 Treatment (Armed Drones, Substitute for Human Judgment):** “In recent years, advancements in Artificial-Intelligence (AI) have led to the emergence of new technologies. One notable AI-enabled technology is **armed drones**. The goal of this AI-enabled technology is to **substitute for human judgment** in this setting.”

**Control Scenario (No Variation in domain and intended purpose):** “In recent years, advancements in Artificial-Intelligence (AI) have led to the emergence of new technologies.”

**6. Adjudicating support, trust, and understanding:**

*Programming Note: As per our 3x2 factorial and between-subject survey experiment design, respondents will view one of the five scenarios, ordered randomly, and then answer several questions*.

1. Do you ***support*** the use of AI under these circumstances? (Q7)

- Strongly Agree (5)
- Agree (4)
- Neither Agree Nor Disagree (3)
- Disagree (2)
- Strongly Disagree (1)

1. Do you ***trust*** the use of AI under these circumstances? (Q8)

- Strongly Agree (5)
- Agree (4)
- Neither Agree Nor Disagree (3)
- Disagree (2)
- Strongly Disagree (1)

1. To what extent do you agree with the following statement: “I have sufficient understanding of AI and how it works across domains.” (Q9)

- Strongly Agree (5)
- Agree (4)
- Neither Agree Nor Disagree (3)
- Disagree (2)
- Strongly Disagree (1)

**7. Open-ended questions for experiment one:**

1. What factors did you consider while evaluating your ***support*** for the AI-enabled technology? Please be as detailed as possible. (Q10)
2. What factors did you consider while evaluating your ***trust*** for the AI-enabled technology? Please be as detailed as possible. (Q11)

**8. Follow-up questions on mediators:**

I will now ask you some questions to get your thoughts on AI-enabled technologies.

*Programming Note: The following questions (6a-6g) are randomized.*

a. To what extent do you agree with the following statement? “Others are using this AI-enabled technology, so not using it means the prospect of missing out.” (Q12—Affect)

- Strongly Agree (5)
- Agree (4)
- Neither Agree Nor Disagree (3)
- Disagree (2)
- Strongly Disagree (1)

b. To what extent do you agree with the following statement? “This AI-enabled technology introduces risks to society but is likely to lead to more benefits overall.” (Q13—Calculation)

- Strongly Agree (5)
- Agree (4)
- Neither Agree Nor Disagree (3)
- Disagree (2)
- Strongly Disagree (1)

c. To what extent do you agree with the following statement? “This AI-enabled technology can substitute and exceed/excel that of humans doing the same task.” (Q14—Efficiency/Substitution)

- Strongly Agree (5)
- Agree (4)
- Neither Agree Nor Disagree (3)
- Disagree (2)
- Strongly Disagree (1)

d. To what extent do you agree with the following statement? “People may be vulnerable to the risks of this technology now, but safety features are improving in ways that reduce their vulnerability.” (Q15—Optimism Bias)

- Strongly Agree (5)
- Agree (4)
- Neither Agree Nor Disagree (3)
- Disagree (2)
- Strongly Disagree (1)

e. To what extent do you agree with the following statement? “People can opt-out of the AI-enabled technology, so they can minimize any associated risk.” (Q16—Opt-Out Capacity).

- Strongly Agree (5)
- Agree (4)
- Neither Agree Nor Disagree (3)
- Disagree (2)
- Strongly Disagree (1)

f. To what extent do you agree with the following statement? “More information about the AI-enabled technology, such as the decision-making algorithm, will make the technology more attractive to use.” (Q17—Transparency).

- Strongly Agree (5)
- Agree (4)
- Neither Agree Nor Disagree (3)
- Disagree (2)
- Strongly Disagree (1)

g. To what extent do you agree with the following statement? “As AI-enabled technology becomes a common part of daily life, society’s acceptance of it will increase.” (Q18—Dependency).

- Strongly Agree (5)
- Agree (4)
- Neither Agree Nor Disagree (3)
- Disagree (2)
- Strongly Disagree (1)

**9. Follow-up demographic and dispositional:**

*Programming Note: The following questions (10a-10f) are randomized.*

a. What is your annual income? (Q19)

- Less than $10,000 (1)
- $10,000 to $25,000 (2)
- $25,000 to $50,000 (3)
- $50,000 to $75,000 (4)
- $75,000 to $100,000 (5)
- $100,000 or more (6)

**(The following questions are designed to assess respondents’ conservativism.)**

b. Generally speaking, do you usually think of yourself as a…? (Q20)

- Democratic (1)
- Independent (2)
- Republican (3)
- Other (4)
- I am not sure (5)

c. Generally speaking, do you think of yourself as…? (Q21)

- Extremely Liberal (1)
- Liberal (2)
- Slightly Liberal (3)
- Moderate, middle of the road (4)
- Slightly Conservative (5)
- Conservative (6)
- Extremely Conservative (7)
- I am not sure (8)

**(The following questions are designed to assess respondents’ religiosity.)**

d. How would you describe your religious affiliation today? (Please select only one) (Q22)

- Protestant Christian (1)
- Catholic (2)
- Other Christian (3)
- Jewish (4)
- Muslim (5)
- Buddhist (6)
- Hindu (7)
- Atheist (8)
- No formal religious affiliation (9)
- Other (10)

**(The following questions are designed to assess respondents’ military status.)**

e. Have you ever served or are you currently serving in the US military (including any

component—Active Duty, National Guard, Reserves)? (Q23)

- Yes (1)
- No (2)

f. In what state do you currently reside? (Q24)

- Alabama (1) … (50)
